# Cells change their sensitivity to an EGF morphogen gradient to control EGF-induced gene expression

**DOI:** 10.1038/ncomms8053

**Published:** 2015-05-11

**Authors:** Jeroen Sebastiaan van Zon, Simone Kienle, Guizela Huelsz-Prince, Michalis Barkoulas, Alexander van Oudenaarden

**Affiliations:** 1 Departments of Physics and Biology and Koch Institute for Integrative Cancer Research, Massachusetts Institute of Technology, Cambridge, Massachusetts 02139, USA; 2 FOM Institute AMOLF, Science Park 104, 1098 XG Amsterdam, The Netherlands; 3 Institut de Biologie de l’Ecole Normale Supérieure, CNRS-Inserm-ENS, 46 rue d’Ulm, 75230 Paris cedex 05, France; 4 Department of Life Sciences, Imperial College London, London SW7 2AZ, UK; 5 Hubrecht Institute, Royal Netherlands Academy of Arts and Sciences and University Medical Center Utrecht, Uppsalalaan 8, 3584 CT Utrecht, The Netherlands

## Abstract

How cells in developing organisms interpret the quantitative information contained in morphogen gradients is an open question. Here we address this question using a novel integrative approach that combines quantitative measurements of morphogen-induced gene expression at single-mRNA resolution with mathematical modelling of the induction process. We focus on the induction of Notch ligands by the LIN-3/EGF morphogen gradient during vulva induction in *Caenorhabditis elegans*. We show that LIN-3/EGF-induced Notch ligand expression is highly dynamic, exhibiting an abrupt transition from low to high expression. Similar transitions in Notch ligand expression are observed in two highly divergent wild *C. elegans* isolates. Mathematical modelling and experiments show that this transition is driven by a dynamic increase in the sensitivity of the induced cells to external LIN-3/EGF. Furthermore, this increase in sensitivity is independent of the presence of LIN-3/EGF. Our integrative approach might be useful to study induction by morphogen gradients in other systems.

## Abstract (web summary)

How morphogen gradients induce complex gene expression programmes in the receiving cells is poorly understood. Here the authors use a quantitative approach to show that in *C. elegans* vulva induction cells control morphogen-induced gene expression by changing their sensitivity to the morphogen.

How cells in developing embryos interpret external signals to make robust cell fate decisions is still an open question. This is particularly challenging for the induction of spatial cell fate patterns by morphogen gradients, where induced cells do not just respond to the absence or presence of a signal, but rather to its exact local concentration^1^,^2^,^3^,^4^. In addition, it is increasingly clear that morphogen gradients can induce complex dynamic gene expression programmes in the receiving cells, which can depend both on the strength and the duration of the morphogen signal^5^. Understanding how the quantitative information contained in morphogen gradients, coupled with its read-out by the downstream gene regulatory network, generates such dynamics is challenging. Quantitative analysis of the induced gene expression dynamics can provide novel insights into these questions. In particular, quantitative measurements can be used to test and constrain mathematical models of the underlying gene regulatory network. In this way, one can identify in a systematic manner which parameters in the model are essential to explain the observed dynamics. Here, we use such a quantitative approach to study *C. elegans* vulva induction, a classical model of spatial cell fate patterning by a morphogen gradient.

The *C. elegans* vulva is induced from six equivalent vulva precursor cells (VPCs): P3.p-P8.p (Fig. 1a). Guided by a spatial LIN-3/EGF gradient from the anchor cell (AC), each VPC adopts 1°, 2° or 3° fate, in a precise spatial pattern: P6.p, the cell closest to the AC, assumes 1° fate, the neighbouring VPCs P5.p and P7.p assume 2° fate and the remaining VPCs assume 3° fate^6^. This cell fate pattern is thought to be established in the following manner: activation of the EGF receptor (EGFR) LET-23 by LIN-3 induces EGF/Ras signalling and subsequent upregulation of Notch ligands *lag-2*, *apx-1* and *dsl-1* in each VPC in a graded manner, depending on the external LIN-3 concentration^7^. Next, the Notch ligands stimulate lateral Notch signalling via the Notch receptor LIN-12, resulting in the inhibition of EGF/Ras signalling in neighbouring VPCs^8^. As a consequence, the higher initial level of Notch ligands in P6.p eventually leads to full inhibition of EGF/Ras signalling in P(5,7).p. In this way, the external LIN-3 gradient is amplified into an all-or-nothing difference in signalling between VPCs, with EGF/RAS signalling and Notch ligand expression restricted to P6.p (1° fate) and high LIN-12/Notch activity in P(5,7).p (2° fate).

The best-studied example of downstream gene expression induced during vulva development is the 1° fate marker *egl-17*, a target of the Ras pathway but otherwise not involved in vulva induction. During early induction, a reporter for *egl-17* was induced by LIN-3 in a graded manner, that is, decreasing with distance from the AC, in P(5,6,7).p, before its expression was later restricted to P6.p^8^. However, it is currently not clear what Notch ligand expression dynamics is induced by LIN-3, despite the pivotal role of Notch ligands in vulval cell fate patterning. Previous experiments showed expression of reporters for all three Notch ligands in P6.p during vulva induction, but were limited in terms of quantification and time resolution^7^. Specifically, it remains unclear whether Notch ligands show a graded expression pattern during early induction, as was observed for *egl-17*, or exhibit different dynamics.

To address these questions we systematically quantify Notch ligand expression during vulva induction. We find that Notch ligands are initially expressed at low levels in multiple VPCs but are expressed at high levels and only in P6.p at late induction. Furthermore, we find that this increase in expression level is due to the VPCs increasing their sensitivity to LIN-3/EGF over time and that this change in sensitivity is independent of LIN-3/EGF and Ras signalling.

## Results

### Dynamic Notch ligand expression during vulva induction

**Figure 1:**
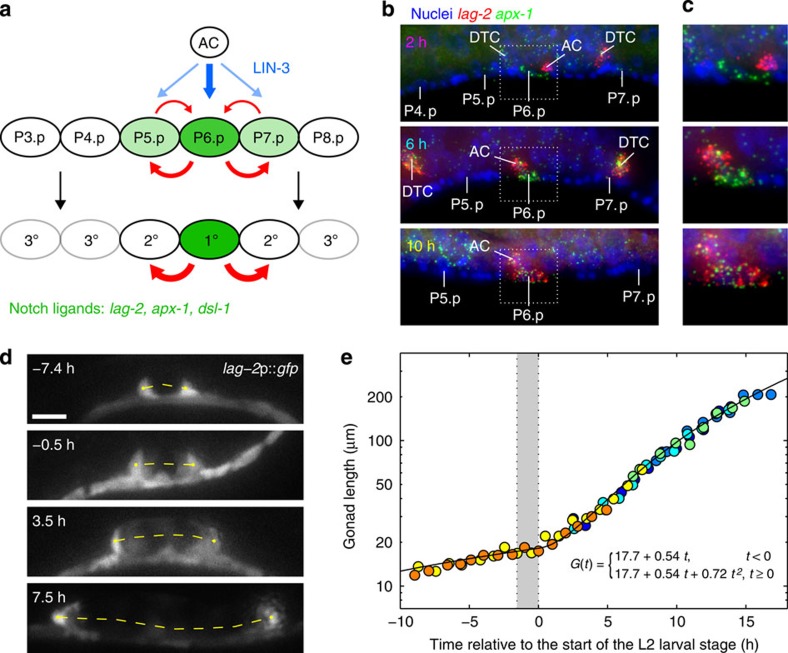
Measuring Notch ligand expression dynamics during vulva induction. (**a**) Schematic representation of vulva induction and vulva precursor cell (VPC) fate specification. A graded LIN-3 signal from the anchor cell (AC, blue) induces EGF/Ras signalling and Notch ligand expression (green) in a spatially graded manner in the VPCs, labelled P3.p-P8.p. Notch ligands stimulate lateral LIN-12/Notch signalling (red arrows) leading to inhibition of EGF/Ras signalling in neighbouring VPCs. As a result, EGF/Ras signalling and Notch ligand expression is restricted exclusively to P6.p which then assumes 1° fate, whereas activated LIN-12 in P5.p and P7.p induces 2° fate. The remaining 3° VPCs do not assume vulval fates. (**b**) Visualization of single-mRNA molecules of the Notch ligands *lag-2* (red) and *apx-1* (green) by single-molecule fluorescence *in situ* hybridization (smFISH) in fixed wild-type (N2) animals at different stages of vulva induction. Nuclei are stained by DAPI (blue). VPCs, the AC and the distal tip cells (DTCs) are labelled. Time is indicated in hours with respect to the start of the L2 larval stage, as determined by gonad length. Scale bar, 10 μm. (**c**) Detail of *lag-2* and *apx-1* mRNA expression in P6.p, corresponding to the dashed boxes in panel (**b**). (**d**) Gonad length extension as measured by the distance between the two DTCs (yellow markers) along the A-P axis (dashed yellow line) in a live animal carrying a *lag-2*p::*gfp* reporter (*qIs56*) as a DTC marker. Scale bar, 20 μm. (**e**) Gonad length as a function of time relative to the start of the L2 larval stage(*n*=5 animals). Each colour corresponds to a single animal followed for a period between 10 and 17 h. The shaded region corresponds to the L1 lethargus. The black line shows the best fit *G*(*t*) to the combined data points of all animals.

We used single molecule fluorescence *in situ* hybridization (smFISH)^9^,^10^ to visualize and count single mRNA molecules of the Notch ligands *lag-2*, *apx-1* and *dsl-1* in individual VPCs in fixed wild-type (N2) animals (Fig. 1b,c). We observed expression in VPCs of *lag-2* and *apx-1* but not *dsl-1* (Supplementary Fig. 1), although there is genetic evidence for a role for *dsl-1* in vulva induction^7^,^11^. We observed *dsl-1* expression in embryos and also in VPCs when overexpressed from a vulva-specific promoter but not when overexpressed from the *dsl-1* promoter (Supplementary Fig. 1). This suggested that *dsl-1* is expressed in VPCs at low levels or inaccessible to our smFISH probes and hence we excluded *dsl-1* from our analysis.

We tested whether the smFISH probes specifically labelled *apx-1* and *lag-2* transcripts. First, we induced *apx-1* RNAi knockdown by feeding RNAi and observed, by smFISH, significant reduction of expression of *apx-1* but not *lag-2* (Supplementary Fig. 2a,b). We did not observe a decrease in *lag-2* level upon *lag-2* RNAi, likely reflecting the variability of RNAi treatment^12^. However, the *lag-2* expression pattern outside of the VPCs was different from *apx-1*, showing expression in the AC and distal tip cells (DTCs) (Fig. 1b,c), which are known to express *lag-2* (refs 13, 14, 15). Finally, expression of both *lag-2* and *apx-1* was absent from VPCs in most animals in a *lin-3(e1417)* mutant where *lin-3* expression in the AC and induction of vulval cell fate are strongly reduced^16^ (Supplementary Fig. 2c,d). Together, these results indicated that the *lag-2* and *apx-1* expression patterns, as observed by smFISH, reflected their induction by LIN-3.

**Figure 2:**
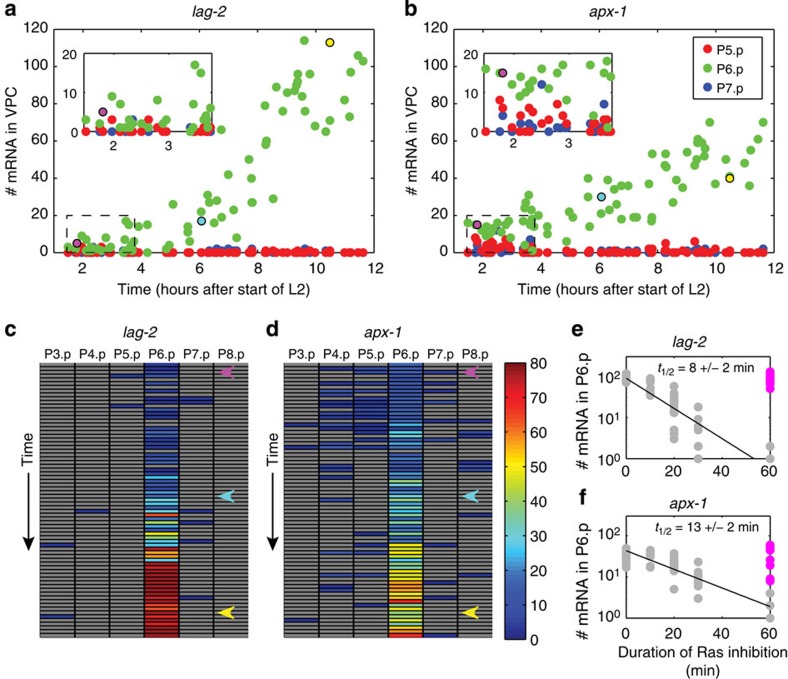
Notch ligand expression dynamics in VPCs during vulva induction in wild-type animals. (**a**) *lag-2* and (**b**) *apx-1* mRNA levels in P5.p, P6.p and P7.p as a function of time relative to the start of the L2 larval stage (*n*=73 animals). This time range extended from the specification of the AC, at ∼0 h, to the first VPC divisions, at ∼12 h. The magenta, cyan and yellow markers indicate levels in P6.p of the animals in Fig. 1b. (Inset) Detail of *lag-2* and *apx-1* mRNA levels during early induction, corresponding to the dashed box in the main figure. (**c**) Overview of *lag-2* and (**d**) *apx-1* expression levels in P3.p-P8.p. Columns correspond to individual VPCs. Rows represent different animals and are sorted according to increasing gonad length. Arrows indicate the animals in Fig. 1b. (**e**) Decay of *lag-2* and (**f**) *apx-1* mRNA levels in P6.p as a function of time after inhibition of EGF/Ras signalling at the late-induction stage (10–12 h, *n*=55 animals). Black lines indicate an exponential fit with half-life *t*_1/2_. Inhibition is achieved by a 25 °C heat shock in a temperature-sensitive *sos-1* mutant. Magenta markers show the expression levels of wild-type animals subjected to the same heat-shock treatment (*n*=13 animals).

As smFISH requires fixation of the sample, each animal studied provided a snapshot of Notch ligand expression at a particular stage of development. To extract time dynamics from such measurements, we determined the gonad length, defined as the distance between DTCs, using the smFISH *lag-2* signal as DTC marker^14^. During vulva induction, gonad length increases over time^17^. We measured gonad length extension as a function of time in live animals carrying a *lag-2p::GFP* transgene as a DTC marker (Fig. 1d,e). We could fit the gonad length *G* as a function of time using the expression *G*(*t*)=17.7+0.54*t* for *t*<0 and *G*(*t*)=17.7+0.54*t*+0.72*t*^2^ for *t*≥0, where *G* is in units of μm, *t* in units of hours and *t*=0 corresponds to the start of the L2 larval stage. Using *G*(*t*) we could then quantify *lag-2* and *apx-1* expression dynamics as a function of time (Fig. 2a,b).

We found that Notch ligand expression occurred in two stages; an early stage (0–4 h, Fig. 1b,c, upper panel, Fig. 2a–d) in which *apx-1* was expressed at ∼15 molecules in P6.p and ∼5 molecules in P(5,7).p. Surprisingly, at this stage we also observed low-level *apx-1* expression in P(3,4,8).p (Fig. 2d), whereas previous experiments suggested that the LIN-3 gradient did not extend beyond P(5,7).p^8^. During this stage *lag-2* expression in P6.p was 
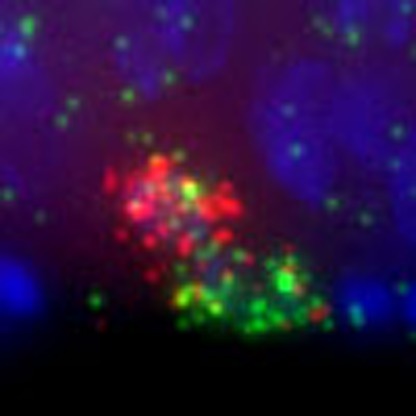
5 molecules and absent from most P(5,7).p cells. During the late stage (4–12 h, Fig. 1b,c, lower panel, Fig. 2a–d), *apx-1* and *lag-2* expression levels increased rapidly in P6.p, with *lag-2* levels two-fold higher than *apx-1* levels. At this stage, *apx-1* expression disappeared from the P(5,7).p cells. In contrast to *apx-1*, *lag-2* expression was almost exclusively restricted to P6.p at all stages (Fig. 2c). Both the wide range of early *apx-1* expression and the later rise in expression levels have not been observed previously using transcriptional reporters^7^,^13^. The first observation provides the best evidence to date that LIN-3 forms a long-range gradient that reaches VPCs as distant as P3.p.

### Conserved expression dynamics in highly divergent isolates

**Figure 3:**
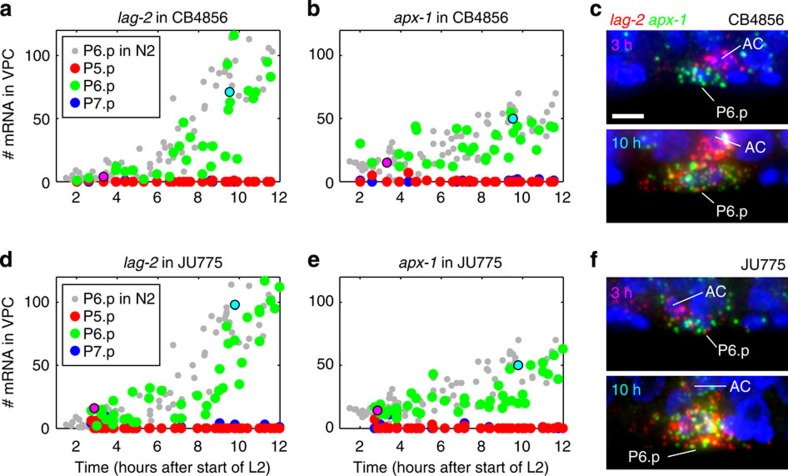
Notch ligand expression dynamics during vulva induction in wild *C. elegans* isolates. (**a**) *lag-2* and (**b**) *apx-1* mRNA levels in P5.p, P6.p and P7.p as a function of time in the wild *C. elegans* isolate CB4856 (*n*=32 animals). Shown in comparison are mRNA levels in P6.p in N2 animals (grey markers). The magenta and cyan markers indicate the mRNA levels in P6.p in the animals in panel (**c**). To facilitate comparison with the data in Fig. 2, we used the expression in Fig. 1e for N2 animals to convert gonad length into time. (**c**) Visualization of single-mRNA molecules of *lag-2* (red) and *apx-1* (green) in P6.p in CB4856 animals at the early (top) and late (bottom) vulva induction stage. Scale bar, 3 μm. (**d**) *lag-2* and (**e**) *apx-1* mRNA levels for the wild *C. elegans* isolate JU775 (*n*=44 animals). (**f**) Visualization of *lag-2* (red) and *apx-1* (green) mRNA molecules in P6.p in JU775 animals.

A surprising feature of the Notch ligand expression dynamics was the abrupt increase in expression levels during late vulva induction (Fig. 2a,b). To examine whether this rise in expression is significant for vulva induction, we used smFISH to visualize *lag-2* and *apx-1* expression in two wild *C. elegans* isolates, CB4856 and JU775, that are highly divergent from the laboratory strain N2 (ref. 18). For both isolates, we observed a clear transition from low to high Notch ligand expression in P6.p (Fig. 3). Surprisingly, despite small differences, many features of the observed expression dynamics were conserved between N2, CB4856 and JU775. In particular, we observed that in all three cases during early induction *apx-1* was expressed more highly than *lag-2*, whereas after the transition to high Notch ligand expression that situation was reversed. In addition, the timing of Notch ligand expression dynamics, as quantified by gonad length extension, was similar between the three strains. Together, the observed similarities suggest that these features of Notch ligand expression are important for vulva induction.

### Expression dynamics is driven by EGF/Ras signalling

Next, we examined whether the increase in Notch ligand expression during vulva induction reflected an integration of the LIN-3 signal, for example, by steady accumulation of long-lived mRNA molecules, or corresponded instead to an instantaneous read-out of the external LIN-3 level. We inhibited the EGF/Ras signalling pathway using a temperature-sensitive *sos-1* mutant, where at 25 °C EGF/Ras signalling is inhibited^19^. We shifted *sos-1(ts)* animals in the late-induction stage (10–12 h) to 25 °C for different time intervals, and measured the *lag-2* and *apx-1* mRNA level in P6.p at the end of the interval. We found that in the absence of EGF/Ras signalling *lag-2* and *apx-1* mRNA levels decayed rapidly, with half-lifes of ∼10 min (Fig. 2e,f). We observed no decay in wild-type animals that underwent the same treatment (Fig. 2e,f and Supplementary Fig. 2e,f). Hence, EGF/Ras signalling dynamics and mRNA turnover are approximately in steady state on the timescale of vulva induction and Notch ligand expression forms a nearly instantaneous read-out of EGF/Ras signalling and the external LIN-3 signal.

During normal development Notch ligand induction is controlled by a combination of the LIN-3 signal and lateral Notch inhibition. Hence, the observed expression dynamics in P6.p could be driven by EGF/Ras signalling in P6.p and/or by (the relief of) inhibitory Notch signalling from P(5,7).p. To decouple these contributions, we measured Notch ligand expression in a Notch receptor mutant, *lin-12(lf)*, where Notch signalling between VPCs is lost^20^. We choose this approach over vulva-specific *lin-12* RNAi, where inhibition of Notch signalling occurs with variable efficiency^21^. However, lack of Notch signalling in *lin-12(lf)* animals typically leads to two ACs^20^, which likely increases the concentration and range of the LIN-3 gradient. Surprisingly, we found that in *lin-12(lf)* animals Notch ligand expression dynamics in P6.p was very similar to wild type, despite the expected accompanying increase in LIN-3 dosage (Supplementary Fig. 3b,c), an observation addressed further below.

**Figure 4:**
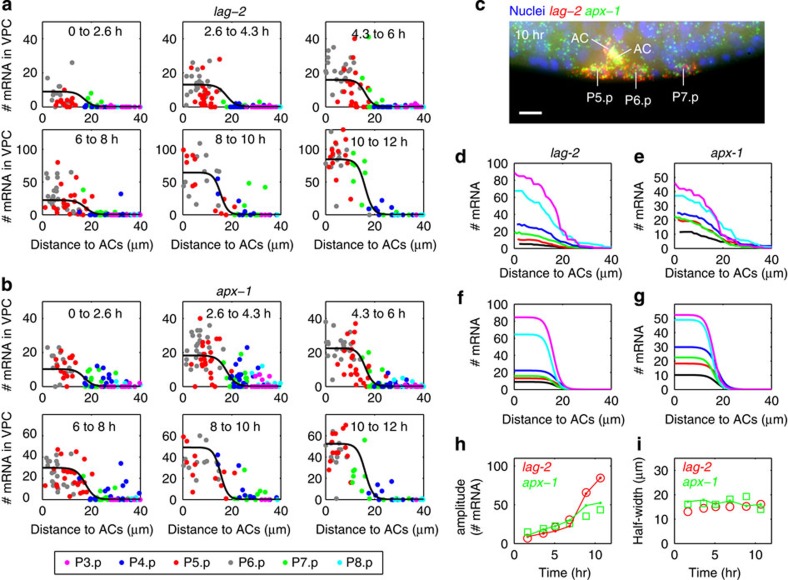
Graded Notch ligand expression dynamics in *lin-12*/Notch mutant animals. (**a**) Level of *lag-2* mRNA in all VPCs as function of the distance of each VPC to the ACs for different stages of induction as characterized by gonad length (*n*=111 animals). Markers indicate expression levels in P3.p (magenta), P4.p (blue), P5.p (red), P6.p (grey), P7.p (green) and P8.p (cyan). Lines indicate best fit of equation (3) to the data. (**b**) Same as in (**a**) but for *apx-1*. (**c**) Visualization of individual *lag-2* (red) and *apx-1* (green) mRNA molecules in a *lin-12(lf)* mutant, showing high Notch ligand expression in P5.p, P6.p and P7.p during late vulva induction. Scale bar, 5 μm. (**d**,**e**) Sliding averages of the expression level of *lag-2* (**d**) and *apx-1* (**e**) for 0–2.6 h (black), 2.6–4.3 h (red), 4.3–6 h (green), 6–8 h (blue), 8–10 h (cyan) and 10–12 h (magenta). Sliding averages were calculated with window size of 5 μm. (**f**,**g**) Best fit of the model in equation (3) to the different time points in panels **a** and **b**. (**h**) Amplitude as a function of time of the *lag-2* (red) and *apx-1* (green) spatial expression profile for the experimental data in panels (**a**) and (**b**) (markers) and the fit to equation (3) (dots). For the experimental data, the amplitude is calculated by an average over the expression levels in all VPCs within a distance <5 μm of the AC. (**i**) Half-width of the *lag-2* (red) and *apx-1* (green) expression profile for the data in (**a**,**b**) (markers) and the fit to equation (3) (dots). The experimentally observed half-width is calculated from the sliding average in (**d**,**e**).

In *lin-12(lf)* animals, *lag-2* and *apx-1* were expressed in multiple VPCs, with high expression levels in P5.p, P6.p and in ∼50% of P7.p at the late vulva induction stage (Fig. 4c and Supplementary Fig. 3a), consistent with the cell fate transformations observed in *lin-12(lf)* animals^22^ and likely due to the increased AC number^21^. We found that Notch ligand expression depended strongly on the distance from the ACs, the source of the LIN-3 gradient, presumably reflecting the shape of the external LIN-3 gradient. To quantify this effect, we plotted the *lag-2* and *apx-1* mRNA levels in each VPC versus its distance along the anteroposterior axis to the centre of the two ACs, which cluster closely together in the gonad (Fig. 4a,b,d,e). We found that *lag-2* and *apx-1* expression levels decreased monotonically with distance from the ACs, with an ∼15–20 μm half-width (Fig. 4d,e,i). We observed that the expression dynamics of *lag-2* and *apx-1* shared characteristics between wild-type (Fig. 2a,b) and *lin-12(lf)* animals (Fig. 4a,b,d,e and Supplementary Fig. 3a–c): in both cases, *apx-1* was expressed earlier and more widely than *lag-2* during the early-induction stage (0–4 h), whereas *lag-2* and *apx-1* expression levels increased markedly in the late stage (10–12 h), with *lag-2* expression levels rising above *apx-1* levels. Hence, these aspects of Notch ligand expression were controlled exclusively by EGF/Ras signalling.

### Mathematical modelling of expression dynamics

We then used mathematical modelling to systematically examine how the observed transition in Notch ligand expression is regulated by EGF/Ras signalling. Previous models focused on crosstalk between the EGF/Ras and Notch pathways in cell fate assignment^23^,^24^,^25^. Here, we focus purely on Notch ligand induction by the LIN-3 gradient and EGF/Ras pathway, without Notch signalling. The signalling network underlying vulva induction, as deduced from genetics experiments, is highly complex^6^,^26^. Explicitly modelling this full complexity would yield a model whose parameters we cannot constrain with our experimental data. Instead, we searched for the simplest model that could reproduce the experimental data for wild-type animals and key mutants. For this, we used the following approach: first, we identified simple candidate models, based on the current knowledge of EGF/Ras signalling^26^,^27^,^28^ and Notch ligand induction^13^, that reproduced the transition in Notch ligand expression in wild-type animals. Then, by comparing the predictions of the different candidate models for key mutants we systematically eliminated all but a single model, strongly constraining the possible mechanisms underlying the transition.

**Figure 5:**
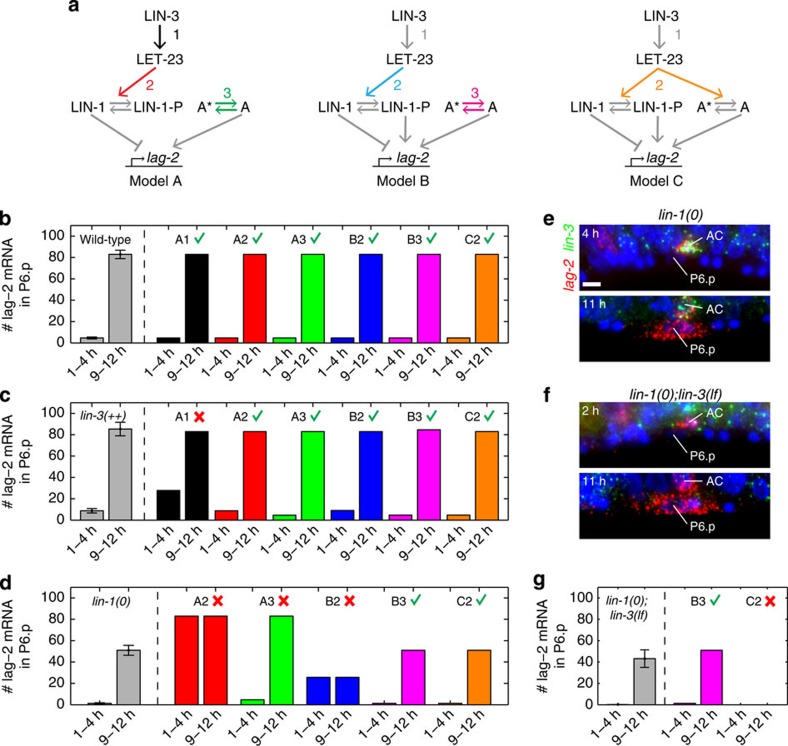
Mathematical modelling of induction of Notch ligand expression. (**a**) Overview of the different models of the EGF/Ras pathway considered in explaining the transition in *lag-2* expression, taking into account the ligand LIN-3, receptor LET-23, the transcription factor LIN-1 and activator A. In model B, but not Model A, LIN-1-P activates *lag-2* expression. In Model C, but not Models A and B, LIN-1 and the activator A are both controlled by Ras signalling. In the models, the change in *lag-2* expression is controlled either by a change in LIN-3 level (Models A1-C1), a change in sensitivity of the Ras pathway to LIN-3 (Models A2-C2) or a change in the amount of activator A (Models A3-B3). As Models A1-C1 are similar, here only Model A1 is considered. See Supplementary Fig. 5 for results on all models. (**b**) Early (1–4 h) and late (9–12 h) *lag-2* expression levels in wild-type animals (grey), with *n*≥24 animals for each time point. Error bars here and in panels (**c**,**d**) and (**g**) indicate s.e.m. Coloured bars show best fits to the wild-type data for Models A1-C2. All models are able to reproduce the data (green tick marks). (**c**) Early and late *lag-2* expression levels in the *lin-3(++)* mutant for the experiment (grey bars, *n*≥8 animals) and best fits of Models A1-C2 (coloured bars). Model A1 failed to fit the experimental data (red cross). (**d**) Same as (**e**) but in the *lin-1(0)* mutant (*n*≥22 animals). Only models B3 and C2 could reproduce the experiment. (**e**) Visualization of *lag-2* (green) and *lin-3* (red) mRNA molecules in P6.p and the AC at early (upper panel) and late (lower panel) vulva induction in the *lin-1(0)* mutant, showing *lag-2* and *lin-3* expression in the AC. Scale bar, 3 μm. (**f**) Same as (**e**) but in the *lin-1(0)*;*lin-3(e1417)* mutant. A transition in *lag-2* expression is visible without *lin-3* expression in the AC. (**g**) Early and late *lag-2* expression in the *lin-1(0)*; *lin-3(e1417)* mutant for the experiment (*n*≥7 animals) and best fit for Models B3 and C2. Only Model B3 was consistent with the experimental data.

We based our models on the following observations: LIN-1 represses *lag-2* expression in VPCs and inhibition of the repressive action of LIN-1 by EGF/Ras signalling is responsible for inducing *lag-2* in P6.p^13^. However, there is evidence that LIN-1 can also activate gene expression^29^,^30^,^31^. Finally, on inhibition of LIN-1 repression, *lag-2* expression is induced by one or more unknown activators^13^. This led us to consider three different models of increasing complexity. In the simplest model (Model A, Fig. 5a), EGF/Ras signalling stimulates the transition of LIN-1 from its unphosphorylated repressive form (LIN-1) to its phosphorylated inactive form (LIN-1-P), after which expression of *lag-2* is induced by the activator A. In this model, three fundamentally different mechanisms could underlie the transition in Notch ligand expression: a change in LIN-3 level during induction (Model A1); a change in sensitivity of the EGF/Ras pathway to LIN-3 (Model A2); or a change in the amount or activity of the activator A (Model A3). The next model (Model B, Fig. 5a) is similar to Model A, but here EGF/Ras signalling stimulates a transition in LIN-1 from a repressive form (LIN-1) to an activating form (LIN-1-P). We assumed that induction of *lag-2* expression occurs at low rate when either LIN-1-P or the activator A are bound to the *lag-2* promoter individually and at a high rate when bound simultaneously. As for Model A, here the transition can be driven by a change in the LIN-3 level (Model B1), in the sensitivity of the EGF/Ras pathway to LIN-3 (Model B2) or in the level of the activator A (Model B3). Finally, we considered a model (Model C, Fig. 5a) where EGF/Ras signalling not only controls the transition between LIN-1 and LIN-1-P but also the amount of activator A. In this case, the transition in Notch ligand expression can be driven by a change in external LIN-3 (Model C1) or in the sensitivity of the Ras pathway to LIN-3 (Model C2).

To calculate expression levels from the models in Fig. 5a, we use the following approach: we assumed that LIN-3 forms a concentration gradient outside the VPCs with its maximum at P6.p. LIN-3 activates the EGFR LET-23 on the VPC surface. The level of EGF/Ras activation in each VPC depends on the total amount of activated EGFR at the VPC surface. The phosphorylation rate of LIN-1 (all models) and the activation rate of the activator A (Model C) depend on the EGF/Ras signalling level. To connect the levels of LIN-1, LIN-1-P and A to *lag-2* expression, we assumed that the *lag-2* transcription rate depends in a model-specific manner on the probability of LIN-1, LIN-1-P and A bound to the *lag-2* promoter (Supplementary Fig. 8). Such ‘thermodynamic models’ of gene expression can successfully reproduce experimental observations^32^,^33^. As a result, we obtained expressions for the *lag-2* transcription rate that depend on five (Model A) or seven parameters (Models B and C). For the full expressions and more details, see the Methods section. Finally, we assumed that all the above processes were in steady state on the timescale of induction and, hence, the models were not explicitly time-dependent. Instead, the expression dynamics in Figs 2, 3, 4, 5 were due to changes in time of the model parameters.

### Dynamics is driven by a change in sensitivity to LIN-3

Notch ligand expression in P6.p appears unaffected by Notch inhibition from P(5,7).p (Supplementary Fig. 3b,c). Therefore, we used the above models, without Notch signalling, to model Notch ligand expression in P6.p in wild-type animals. We tested the ability of all models to reproduce the wild-type data for *lag-2* expression, which showed the strongest transition in expression level. We fitted all models to the *lag-2* expression levels in P6.p for early (1–4 h) and late (9–12 h) induction and each model could correctly reproduce the transition (Fig. 5b and Supplementary Fig. 5). These fits were not unique: many combinations of parameter values reproduced the observed transition.

Next, we tested whether the increase in Notch ligand expression reflected an increase in external LIN-3. The corresponding models (A1, B1 and C1) were very sensitive to changes in LIN-3 level: in general, they predicted significant rises in expression level on increasing LIN-3 dosage. We experimentally increased LIN-3 dosage using a *lin-3(++)* mutant that carried an integrated transgenic array, *syIs1*, that strongly overexpresses *lin-3* in the AC^34^. Surprisingly, in *lin-3(++)* mutants, the *lag-2* expression level in P6.p did not increase, for both early and late induction (Fig. 5c). However, the LIN-3 dosage in this mutant had clearly increased, as we observed a strong increase in *lag-2* expression in all other VPCs (Supplementary Fig. 3b–d). This result was consistent with the observed lack of change in expression in the *lin-12(lf)* mutants that contained an extra AC. All models, even A1, B1 and C1, could reproduce this lack of increased expression at the late stage, if the LIN-3 level at that stage was so high that all EGFRs were saturated by bound LIN-3 and hence further increases in LIN-3 had little effect. However, model A1 could not reproduce the lack of change in expression at the early time point and hence we considered this model inconsistent with the experimental data. We excluded the other LIN-3-dependent models, B1 and C1, for similar reasons (Supplementary Fig. 5). We found the same transition in *lag-2* expression in a *let-60*/Ras gain of function mutant^35^, where Ras signalling is constitutively active in all VPCs (Supplementary Fig. 4e,g). Together, these results showed that the transition in Notch ligand expression was not due to an increase of LIN-3 level or LET-23 activation.

Next, we tested whether the transition in expression level depended on LIN-1. We quantified *lag-2* expression in a mutant, *lin-1(0)*, where vulval cell fate is induced in almost all VPCs, even in animals without an AC^36^. If the change in expression is driven by a change in Ras signalling that exclusively had an impact on LIN-1 and LIN-1-P levels (Model A2 and B2), the *lag-2* expression level at the early- and late-induction time points was predicted to be identical (Fig. 5d). However, if the change in expression is due to an increase in the amount of the activator A either independent (Model A3 and B3) of or dependent on Ras signalling (Model C2), the transition from low to high *lag-2* expression was predicted to occur even without LIN-1 (Fig. 5d). Indeed, in *lin-1(0)* animals we observed a strong rise in *lag-2* expression level in all VPCs, with few differences between VPCs (Fig. 5d, Supplementary Fig. 4a–d). This ruled out Models A2 and B2. Unexpectedly, in *lin-1(0)* animals *lag-2* expression levels never reached the fully induced levels observed for wild-type induction and were overall lower than in wild-type animals. Previous experiments showed that the main role of LIN-1 is to inhibit vulval cell fate^36^ and *lag-2* expression^13^, which is supported by our observation of *lag-2* expression in all VPCs in *lin-1(0)* animals. However, the assumption that LIN-1 only acts as a repressor (Model A3) predicted that absence of LIN-1 would lead to full induction of *lag-2* (Fig. 5d). However, if LIN-1-P also functioned as an activator of *lag-2* expression (Models B3 and C2), we could fit the transition with reduced *lag-2* expression levels. These results provide the most direct evidence so far for an activating role of LIN-1 in *lag-2* expression, perhaps to fine-tune the expression levels.

Finally, we tested whether the change in expression was due to a change in activation that is dependent on the LIN-3 signal and downstream Ras signalling (Model C2) or independent of LIN-3 (model B3). We measured *lag-2* expression dynamics in a *lin-1(0);lin-3(e1417)* mutant. Model C2 predicted strongly reduced *lag-2* expression in the *lin-1(0);lin-3(e1417)* mutant due to the lack of LIN-3 input, similar to our observations in the *lin-3(e1417)* mutant (Supplementary Fig. 2c,d). However, Model B3 predicted that as the change in the amount of activator A occurred independently of the LIN-3 level and Ras signalling, the transition in *lag-2* expression level would remain unchanged compared with the *lin-1(0)* mutant. Indeed, we found that *lag-2* expression in the *lin-1(0);lin-3(e1417)* still showed a clear transition, with the exact levels very similar to what we observed in the *lin-1(0)* animal (Fig. 5g), while the level of *lin-3* in the AC was indeed strongly reduced compared with *lin-1(0)* animals (Fig. 5e,f). Hence, the transition in *lag-2* expression is regulated downstream of LIN-1 in a LIN-3-independent manner, presumably by temporal modulation of the unknown activator(s) of *lag-2* expression.

### Different threshold for *lag-2* and *apx-1* expression

**Figure 6:**
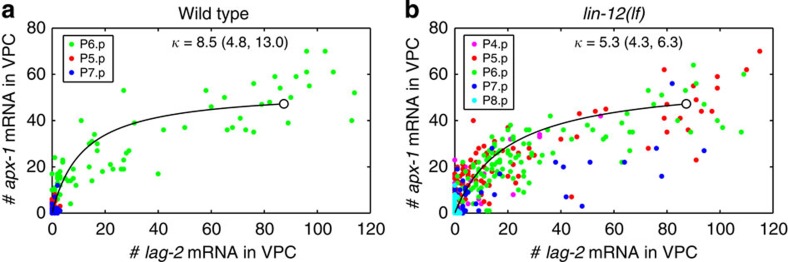
Single-cell correlation in Notch ligand expression. (**a**,**b**) Single-cell correlation between *apx-1* and *lag-2* mRNA level in P(5–7).p in (**a**) wild-type animals (*n*=73) and P(4–8).p in (**b**) *lin-12(lf)* animals (*n*=111) . Line indicates a fit to equation (1) with the value of *κ* as shown in the figure. The values between brackets give the 95% confidence interval for *κ*, as obtained by bootstrapping. The white marker indicates the observed average maximally induced levels 
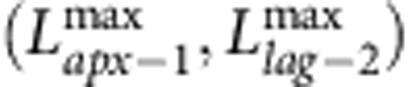
. Owing to the absence of lateral Notch inhibition in *lin-12(lf)* animals, multiple VPCs show high levels of Notch ligand expression.

We found only model B3 consistent with the key mutant data for *lag-2* expression. We then examined whether this model could also explain the full *lag-2* and *apx-1* spatial expression profiles in *lin-12(lf)* animals (Fig. 4). The *apx-1* promoter contains sequences similar to the promoter elements, including LIN-1 binding sites, that control *lag-2* expression in P6.p^13^. Hence, we assumed that *apx-1* is induced as *lag-2*, but with different values for the binding rates of LIN-1, LIN-1-P and the activator A to the *apx-1* promoter. Assuming that *lag-2* and *apx-1* expression are both controlled by the same combination of repressive (LIN-1) and inductive signals (LIN-1-P and A), the extended model B3 predicted that in each VPC the mRNA levels *L*_*apx*−1_ and *L*_*lag*−2_ obeyed the relation:




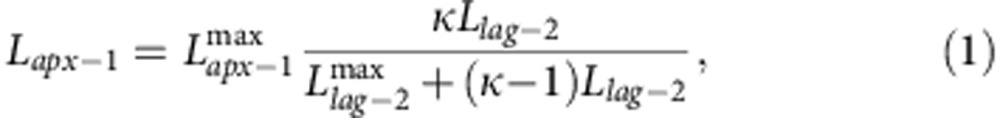




which depends only on the two variables 
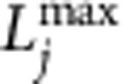
, the maximally induced mRNA level for ligand *j*, and a parameter *κ*, the ratio between the thresholds for induction of *lag-2* and *apx-1* by the activator A. Here *κ*>1 would mean that *apx-1* has a lower threshold to induction by Ras signalling than *lag-2*. (Supplementary Note 8). In agreement with this prediction, we found that for both wild-type and *lin-12(lf)* animals (Fig. 6) the *lag-2* and *apx-1* mRNA levels for all VPCs largely followed the predicted curve. We then used this single-cell data to constrain the parameter κ. First, we measured 
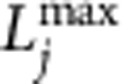
 by averaging mRNA numbers for P6.p in wild-type and *lin-12(lf)* animals at >9 h, yielding 89±3 transcripts for *lag-2* and 53±2 transcripts for *apx-1* (Supplementary Fig. 3e,f), similar to the average mRNA levels in the *lin-3(++)* overexpression mutant (Fig. 5c and Supplementary Fig. 3e,f). We then fitted equation (1) to the experimentally observed correlation data by varying *κ* (Fig. 6 and Supplementary Fig. 6), observing good agreement for *κ*≈5−10, that is, *apx-1* having a 5- to 10-fold lower threshold to induction by EGF/Ras signalling compared with *lag-2*.

Using the measured values of 
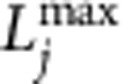
 and *κ*, we were able to find many fits that reproduced both the time dynamics and spatial expression profiles of both *lag-2* and *apx-1* (Fig. 4a,b,f–i) as well as the gene expression dynamics for the *lin-3(++)* and *lin-1(0)* mutants, with parameters constrained to a small region of parameter space (Supplementary Fig. 7). See the Methods section for details of the fitting procedure. All these fits showed the same increase in activator A over the course of induction while the LIN-3 gradient decay length remained approximately constant. In the model, the difference in threshold is sufficient to explain that during the early stage *apx-1* is expressed earlier and more widely than *lag-2* (0–4 h, Fig. 2a–d), even though both are induced by the same signalling pathway.

## Discussion

It is becoming increasingly clear that morphogen gradients can induce complex temporal programmes of gene expression^5^. We quantified gene expression induced by the LIN-3 gradient during vulva induction and found that Notch ligand expression was highly dynamic, with clear differences between *lag-2* and *apx-1* both in timing and spatial extent. In particular, we found that during early vulva induction *apx-1* expression is induced in multiple VPCs, even in P4.p and P8.p, which are far away from the AC, providing the most unambiguous evidence to date that LIN-3 acts as a long-range signal. Subsequently, expression of *lag-2* and *apx-1* became restricted to P6.p, the VPC assuming 1° fate. After this, we observed a striking transition from low to high Notch ligand expression in P6.p. We observed similar dynamics in a mutant lacking lateral Notch signalling, indicating that the relative time and spatial extent of Notch ligand induction as well as the transition in their expression level are regulated by EGF/Ras signalling alone. Finally, we found that the transition in expression level in P6.p was robust to large changes in LIN-3 dosage.

Our experiments provide no conclusive function for the transition in Notch ligand expression in P6.p. However, we do find similar expression dynamics in two divergent wild *C. elegans* isolates, suggesting a significant role for this transition in vulva induction. An intriguing observation is that the rise in Notch ligand expression occurs after *lag-1* and *apx-1* expression has been restricted to a single VPC, a process that is often thought to be the main consequence of Notch signalling during vulva induction. However, Notch signalling acts twice to regulate cell fate decisions during vulva induction: Before the VPC S phase *lin-12* influences a 1° versus non-1° fate decision in P6.p and after S phase induces 2° fate decision in P(5,7).p^37^. The observed timing of the transition in Notch ligand expression suggests that the first decision might occur when Notch ligand expression is low and that the expression levels rise in time for the later decision.

By fitting mathematical models to the experimental results, we systematically tested different potential network topologies of the EGF/Ras signalling network. For each network topology we examined different mechanisms for the observed transition in Notch ligand expression levels. We found that only a single model (Model B3) was consistent with all our experimental observations in different mutants of the EGF/Ras pathway. In this model, the transition in Notch ligand expression is not due to changes in level of the external LIN-3 signal, but instead due to an intrinsic, LIN-3-independent change in sensitivity to external LIN-3. We extended this model to fit expression data for both *lag-2* and *apx-1* by only assuming different rates of binding of the Ras effector LIN-1 and the activator A to their binding sites in the *lag-2* and *apx-1* promoters. Previously, it was found that induction of *lag-2* expression by EGF/Ras signalling was controlled by promoter elements that are also present in the *apx-1* promoter^13^. Our results suggest that the observed differences between *lag-2* and apx-1 expression could be due to small changes in affinity of transcription factors to these promoter elements^38^.

The change in intrinsic sensitivity underlying the transition in Notch ligand expression could have occurred at several levels of the EGF/Ras pathway. For instance, in the model the sensitivity is determined by the level of the EGFR LET-23, the transduction of EGFR activity by EGF/Ras signalling and the threshold of Notch ligand expression to activation by EGF/Ras signalling (Supplementary Notes 1–3). It is known that expression of the EGFR LET-23 increases in P6.p during induction^27^, which would have been a natural mechanism to generate the change in sensitivity to the LIN-3 signal. It is therefore surprising that we still observe a clear transition in Notch ligand expression level in *lin-1(0);lin-3(e1417)* animals, clearly indicating that this transition is independent of the presence of the LIN-3 signal and the EGF/Ras signalling pathway.

Previous experiments have shown that *lag-2* expression not only requires inhibition of the repressive action of LIN-1 by EGF/Ras signalling, but also the action of one or more unknown activators present in all VPCs^13^. Our results show that temporal regulation of these activators might explain the change in sensitivity we observed in the *lin-1(0);lin-3(e1417)* mutant. Our model makes a strong prediction of the expression dynamics on loss of the activator (Supplementary Fig. 5): in this case, Notch ligand expression remains at a low constant level during the entire induction process, reflecting basal induction by the activator LIN-1-P. Similarly, the model predicts that loss of the temporal regulators should result in low, constant Notch ligand expression during induction. In general, the observed dynamic changes in sensitivity point to a so far unrecognized temporal aspect of the response to the external LIN-3 signal. It would be interesting to examine whether the change in sensitivity to LIN-3 of Notch ligand induction is part of a larger temporal programme. For instance, it might be possible that other genes expressed in 1° fate cells, including other Ras targets, might show concomitant changes in expression.

In conclusion, our study showed that during vulva induction downstream gene expression is not controlled exclusively by the external LIN-3 gradient and lateral Notch inhibition but also by the intrinsic modulation of the downstream signal by the receiving cells. To obtain these results we relied crucially on smFISH to quantify with high precision differences in expression levels both between different cells and in time. In addition, even though smFISH requires fixation of the animals, we could still obtain dynamical information by using anatomical markers, in our case gonad length, or by precisely timed perturbations to the signalling network. Finally, we analysed the smFISH data using mathematical models of the signalling network, which proved essential in comparing the different potential mechanisms underlying the observed dynamics. Together, these results show that the combination of quantitative smFISH data and mathematical modelling can be a powerful tool to dissect the dynamics of signalling pathways in development.

## Methods

### *C. elegans* strains and culture

All strains were handled according to the standard protocol^39^. Wild-type nematodes were strain N2. The following mutations and integrated transgenic arrays were used in this study: LG*II*: *rff-3(pk1426)*^40^, LG*III*: *lin-12(n941)*^20^, LG*IV*: *lin-1(n304)*^36^, *lin-3*(e1417)^16^, *eor-1(*cs28)^29^, LG*V*: *sos-1(cs41)*^19^, *qIs56 [lag-2p::gfp; unc-119(+)]*^41^, LG*X: syIs1[lin-3(++); unc-31]*^34^. In addition, we used the following extrachromosal transgenic arrays: *lin-31p::dsl-1*, *dsl-1p::dsl-1*. To create the *lin-1(n304);lin-3(e1417)* double mutant, which is phenotypically similar to the *lin-1(n304)* mutant, we followed the approach in ref. 42. Specifically, we picked *lin-3(e1417)* homozygous animals from the progeny of doubly heterozygous hermaphrodites by selecting those that showed a vulvaless (Vul) phenotype. These animals could be either *lin-1(n304)* heterozygous or *lin-1(+)* homozygous, but not *lin-1(n304)* homozygous, as these would show a multivulva (Muv) phenotype. In the next generation, we obtained the double mutant by selecting animals with a Muv phenotype indicating *lin-1(n304)* homozygosity. In addition, we confirmed by smFISH that in *lin-1(n304);lin-3(e1417)* animals *lin-3* was not expressed in the AC (Fig. 5e,f). All strains were grown at 20 °C. To study the effect of the inhibition of EGF/Ras signalling, *sos-1(cs41)* animals were grown on NGM plates at 20 °C and shifted to 25 °C by moving the plates to a water bath for different periods of time, after which animals were fixed immediately.

To create the *lin-31p::dsl-1* strain (JU2078), a *lin-31::dsl-1(+)::unc-54* construct was injected in N2 at a concentration of 80 ng μl^−1^ in the injection mix. The *lin-31::dsl-1::unc-54* construct was built by cloning the *dsl-1* coding sequence into pB253 [1] as a BglII/NotI fragment using primers dsl-1BglIIF (5′-cgccagatctatgctcaaatatcttatattccttg-3′) and dsl-1NotIR (5′-gctggcggccgcggattcacaatcgaggaagcgt-3′). The *dsl-1p::dsl-1* strain was created by injecting an 8.9-kb PCR product in N2 at a concentration of 30 ng μl^−1^ in the injection mix. This fragment was amplified using primers dsl-1F2 (5′-cgtctgagggaagcaagttc-3′) and dsl-1R2 (5′-agcattcggagagcctgata-3′) and contains the *dsl-1* coding sequence and 7.7 kb upstream to the ATG sequences. In both cases, transgenic animals were identified and maintained following the expression of *myo-2::GFP* in the pharynx.

### Single-molecule fluorescence *in situ* hybridization

Probe design and smFISH hybridization were performed as previously described^9^,^10^ to visualize mRNA transcripts in L2 and L3 larvae. Probes for smFISH hybridization were designed for optimal GC content using a web-based program (www.singlemoleculefish.com) and coupled to Cy5 (GE Amersham) or Alexa594 (Invitrogen). The sequences of the oligonucleotide probes used in this study are listed in Supplementary Data 1. Animals were collected by washing plates with M9 and were fixed in 4% formaldehyde in 1 × PBS for 45 min at room temperature. Fixed animals were permeabilized in 70% ethanol overnight at 4 °C. Subsequently, animals were incubated with the smFISH probes overnight at 30 °C in hybridization solution containing 10% formamide. The next day, animals were washed twice with 10% formamide and 2 × SSC, each time followed by an incubation for 30 min at 30 °C. To visualize cell nuclei, DAPI was added at 5 μg ml^−1^ at the last wash step. Microscopy images were acquired with a Nikon Ti-E inverted fluorescence microscope, equipped with a × 100 plan-apochromat oil-immersion objective and a Princeton Instruments Pixis 1024 CCD camera controlled by MetaMorph software (Molecular Devices, Downington, PA, USA). Exact three-dimensional positions of smFISH fluorescent spots in each animal were detected using a custom MATLAB (The Mathworks) script, based on a previously published algorithm^9^. In brief, we first convolved smFISH microscopy images with a Gaussian filter to increase the brightness of spots of the correct size and suppress the background signal. Next, we select candidate spots by thresholding, using a manually determined threshold. We further refined the candidate spots by finding regional intensity maxima within each candidate spot, to separate smFISH spots whose fluorescence signals are partially overlapping. Finally, the resulting smFISH spots were manually assigned to individual VPCs.

### RNAi by feeding

Bacteria were grown overnight, concentrated by centrifugation and then seeded onto LB plates containing 1 μM IPTG and 50 μg ml^−1^ Ampicillin. We performed *apx-1* RNAi in a sensitized *rrf-3* background^40^, as we observed no knockdown in wild-type animals. Eggs of *rrf-3* animals were collected by bleaching and transferred to fresh RNAi plates. Hatched animals were fixed after ∼30 h for smFISH staining. The *apx-1* RNAi feeding clone was constructed by amplifying a fragment corresponding to *apx-1* from N2 cDNA using primers apx-1F1 (5′-caccatcttcctctgcatca-3′) and apx-1R1 (5′-tttccacacaaatcgcaaaa-3′). This fragment was first cloned into pDONR 221 (Invitrogen) and then transferred to a Gateway compatible L4440 plasmid.

### Timelapse imaging

Individual animals were staged on an agar pad with a small patch of *E. coli* as a food source^43^. In brief, a single L1 animal was transferred to a small drop of M9 on a 5% agar pad. A cover slip was coated with a small amount of OP50 bacteria transferred from a petri dish with a worm pick and then gently placed on top of the agar pad. To prevent the sample from drying out, the coverslip was sealed with VALAP, an equal-weight mixture of vaseline, lanolin and paraffin wax. Staged animals were grown at 20 °C and briefly imaged at ∼1 h intervals at room temperature, using a Nikon Ti-E inverted fluorescence microscope, equipped with a plan-fluor × 40 objective and a Photometrics HQ2 CoolSnap camera, controlled by μManager software (http://www.micro-manager.org).

### Fit of gonad length extension

For each frame, we manually measured the gonad length as the distance along the anteroposterior axis between the two DTCs. Entry into and exit from the L1 lethargus was monitored by the reduction of movement and absence of pharyngeal pumping. We found that the simplest function that reproduced the observed dynamics of gonad length extension had the form *G*(*t*)=*a*+*bt* during the L1 larval stage and *G*(*t*)=*a*+*bt*+*ct*^2^ during the L2 larval stage, where *t*=0 corresponded to the end of L1 and we constrained the parameters *a* and *b* to have identical values in L1 and L2. We defined *G*(0) as the largest observed gonad length for an animal in lethargus. For three animals we started the experiment after the L1 lethargus and hence we did not know the time relative to the start of L2 at the beginning of the experiment. As a result, we had six fit parameters: the three coefficients *a*, *b* and *c* and three time intervals Δ*t*_*i*_, measuring the time between the start of the experiment for that animal and the start of L2. We then obtained values for *a*, *b*, *c* and Δ*t*_*i*_ by minimizing the sum of squares error (SSE) of *G*(*t*) with respect to the measured gonad length extension.

### Mathematical models of Notch ligand induction

Following the approach outlined in the main text, we arrived at the following expressions of the *lag-2* transcription rate *r* for the different networks in Fig. 5a. For Model A:




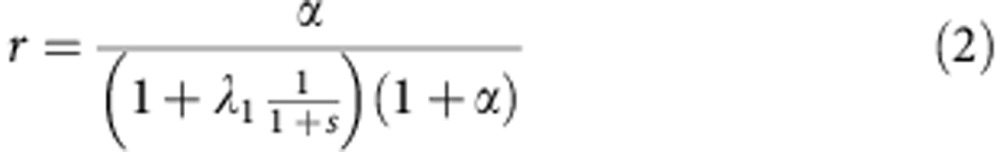




For Model B:




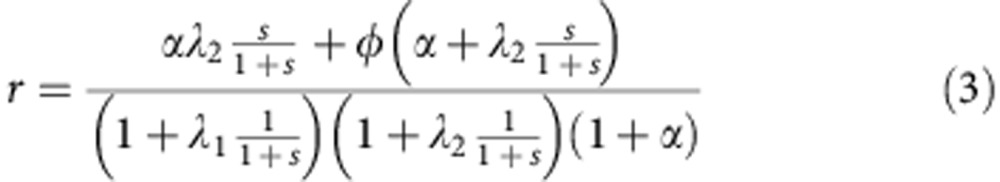




For Model C:




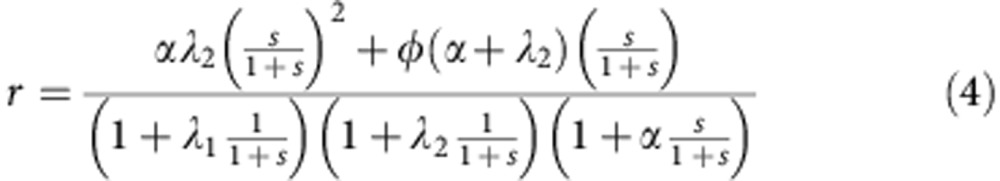




where *λ*_1_ gives the strength of binding of LIN-1 to the promoter, *λ*_2_ the strength of binding of LIN-1-P and *α* the strength of binding of the activator A. The parameter *ϕ*<1 indicates for Models B and C the rate of transcription for LIN-1-P and A bound separately to the promoter, compared with the rate for LIN-1-P and A bound simultaneously, which is set to *r*=1. The parameter *s* represents the strength of Ras signalling and, for a VPC at a distance *x* to the AC, is given by:




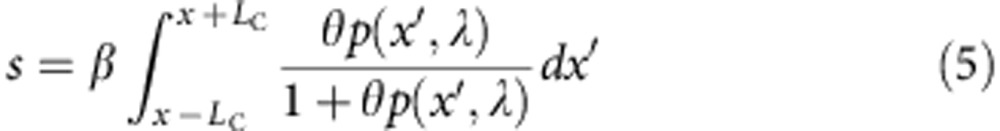




where *β* indicates how strongly Ras signalling is induced by a given LIN-3 input, *θ* indicates the external LIN-3 level, *λ* is the LIN-3 gradient decay length, 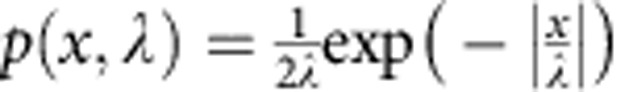 and *L*_C_ is the length of the VPC body along the anteroposterior axis. For a derivation of the above expressions, see Supplementary Notes 1–3. For Models A1, B1 and C1, *θ* is the only parameter that changes in time, whereas the other parameters remain constant. For models A2, B2 and C2, only *β* changes as a function of time, while for models A3 and B3 only *α* changes as a function of time. The models were fitted to the data for the mutants in Fig. 5 as follows: during the fitting procedure we constrained parameter values so that each model exactly reproduced the wild-type data (Fig. 5b) and in addition showed no induction of *lag-2* expression in the absence of LIN-3, that is, *s*≈0. For each combination of parameter values, we calculated the change in expression in the *lin-3(++)* mutant by increasing the LIN-3 dosage tenfold, that is, *θ*′=10*θ*, and in the *lin-1(0)* mutant by setting the total amount of LIN-1 to zero, corresponding to *λ*_1_,*λ*_2_=0. We then found parameter values for which the SSE with respect to the *lin-3(++)* and the *lin-1(0)* mutant data was minimized. If a model was able to reproduce the mutant data, often many combinations of the parameters provided an equally good fit. However, if a model could not produce a good fit to the data, we concluded that it was incorrect or incomplete. Finally, we calculated the expression dynamics for the *lin-1(0); lin-3(e1417)* mutant by simultaneously setting *λ*_1_,*λ*_2_,*s*=0 and the hypothetical mutant lacking the activator A by setting *α*=0. For full details on the fitting procedure, see Supplementary Note 6.

### Fit of single-cell expression correlation

We fitted equation (1) to the experimental data in Fig. 6 by varying the single free parameter κ. We calculated the error by calculating the shortest distance of each data point to the curve of equation (1) and summing the distances of all data points. We did not use the distance along one of the two axes because the deviations from the mean curve are due to independent fluctuations in both *lag-2* and *apx-1* mRNA number. We performed bootstrap analysis by randomly sampling data points from our data set with the same sample size as the original data set and repeating the fitting procedure for each bootstrap sample. The 95% confidence levels reported in Fig. 6 are for 250 bootstrap samples. Distributions of *κ* obtained by bootstrap sampling are shown in Supplementary Fig. 6b,c.

### Fit of spatial expression profiles

We extended model B3 to incorporate both *lag-2* and *apx-1* induction, by making the parameters for binding of LIN-1-P and the activator A dependent on the ligand identity, that is, instead of two parameters *λ*_2_ and α, we now had four parameters *λ*_2_^*i*^ and *α*^*i*^ for each ligand *i*. The remaining parameters, *θ*, *β*, *λ*, *ϕ* and *λ*_1_, were the same for the two ligands. Here, *λ*_2_^*i*^ determines the mRNA level for ligand *i* for maximal induction, that is, *α*^*i*^→∞, whereas *α*^*i*^ controls the actual induction level as a function of time. The number of free parameters of the full model B3 is constrained by the experimental data in the following manner: first, we assumed that the parameter *κ* reflects the relative threshold to induction by the activator A, meaning that *α*^*apx*−1^=*κα*^*lag*−2^. Next, for a fixed value 
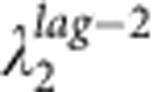
, the value of 
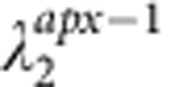
 was completely determined by the ratio of fully induced expression levels 
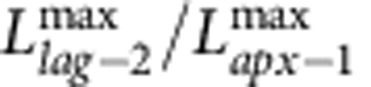
. Finally, the value for *λ*_1_ is determined by the constraint that no induction should occur in the absence of LIN-3, that is, *s*≈0. Hence, we only had to consider four free parameters that were constant in time, *θ*, *β*, *ϕ* and 
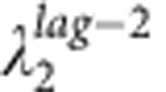
, and two free parameters that potentially varied per time point: *α*^*lag*−2^, the amount of activator A, which mainly determined the amplitude of Notch ligand expression, and *λ*, the LIN-3 gradient decay length, which mainly determined the half-width of the spatial expression profile.

We generated 5 × 10^4^ random parameter combinations of the parameters *θ*, *β*, *ϕ* and 
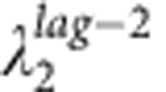
. For each parameter the value was given by 
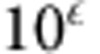
, with the exponent 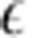 uniformly distributed in a particular interval, so that the parameter values span many orders of magnitudes (Supplementary Fig. 7). For each time point, we obtained the average VPC body length as the mean distance between nuclei of adjacent VPCs for P4.p-P8.p, averaged over all animals in the time point.

This yielded *L*_*C*_={15,15,17,19,19,18}μm for each time point ordered by increasing time of induction. For each random parameter combination and for each time point, we then obtained a LIN-3 decay length and amount of activator A that formed the best fit to the measured spatial expression profiles in Fig. 4a,b. Specifically, we obtained the values for *λ* and *α*^*lag*−2^ that minimized the SSE between the experimental data and the spatial expression profile calculated for Model B3 using equation 3. In general, we found many parameter combinations that were able to reproduce the experimental data in Fig. 4a,b, all constrained to very specific regions in parameter space (Supplementary Fig. 7a). We subsequently selected the subset of these fits that also accurately reproduced the gene expression dynamics for the *lin-3(++)* and *lin-1(0)* mutants (Supplementary Fig. 7b,c). Specifically, for each parameter combination we also calculated the *lag-2* expression dynamics in P6.p, that is, for *x*=0, for both the *lin-3(++)* mutant (*θ*′=10*θ*) and the *lin-1(0)* mutant (*λ*_1_,*λ*_2_=0). To allow for comparison between the SSEs for different mutants and different time points, we normalized each of the different SSEs by the median SSE over all 5 × 10^4^ parameter combinations. Finally, we calculated a single SSE for the fit to the *lag-2* and *apx-1* expression profiles by summing the normalized SSEs for each time point and ligand. This still yielded many good fits, but constrained the parameters to a substantially smaller region of parameter space (Supplementary Fig. 7a). To select the overall best fit, we selected the parameter combination that minimized the sum of the single SSE for the spatial expression profiles and the SSEs with respect to the *lin-3(++)* and the *lin-1(0)* mutants. This fit, which is very similar in quality to the best 1% of fits, accurately captures the observed spatial expression profiles and time dynamics of both *lag-2* and *apx-1* (Fig. 4a,b,f–i).

## Additional information

**How to cite this article:** van Zon, J.S. *et al*. Cells change their sensitivity to an EGF morphogen gradient to control EGF-induced gene expression. *Nat. Commun*. 6:7053 doi: 10.1038/ncomms8053 (2015).

## Supplementary Material

Supplementary Figures and Supplementary Notes Supplementary Figures 1-8, Supplementary Notes 1-10

Supplementary Data 1 The sequences of the oligonucleotide probes
