# Supplementary material for: Cells change their sensitivity to an EGF morphogen gradient to control EGF-induced gene expression
Source: Nat Commun. 2015 May 11;6:7053. doi: 10.1038/ncomms8053 (PMC4438782; doi:10.1038/ncomms8053)
Supplement: Supplementary Figures and Supplementary Notes — Supplementary Figures 1-8, Supplementary Notes 1-10 [file ncomms8053-s1.pdf]

Supplementary figures

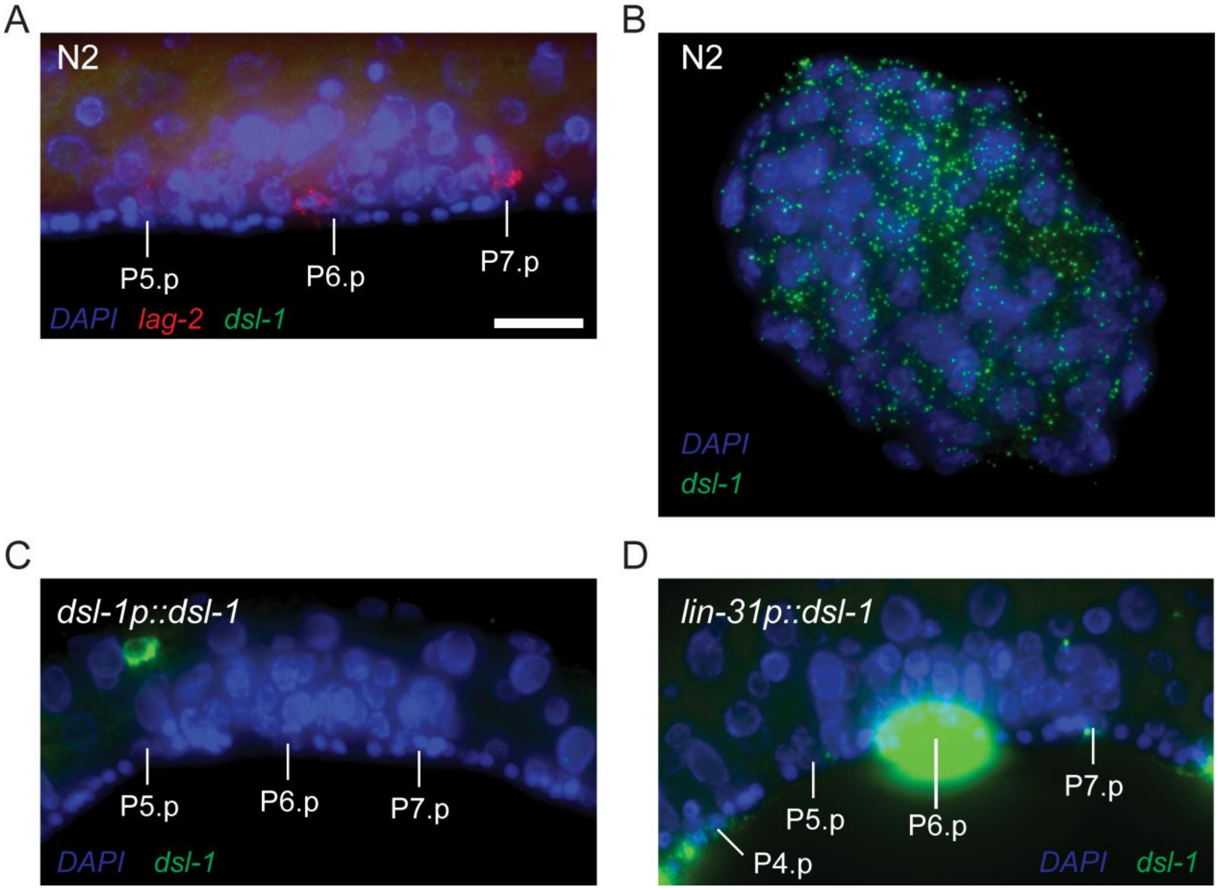

Supplementary Figure 1. **Expression of the Notch ligand *dsl-1* assayed by smFISH.**

(A) Visualization of *lag-2* (red) and *dsl-1* (green) mRNA molecules by smFISH in an N2 animal during vulva induction. The scale bar corresponds to 10μm. **(B)** Expression of *dsl-1* (green) in an N2 embryo. **(C)** Absence of *dsl-1* (green) mRNA in VPCs during vulva induction in *dsl-1p::dsl-1* animals where *dsl-1* is overexpressed from its own promoter. **(D)** Expression of *dsl-1* (green) during vulva induction in *lin-31p::dsl-1* animals, where *dsl-1* is overexpressed from a VPC-specific promoter. Expression in P6.p is too strong for individual smFISH spots to be visible. Individual *dsl-1* mRNA molecules are clearly visible in other VPCs, in particular in P4.p. For all panels, a representative example was selected from $n \geq 3$ animals.

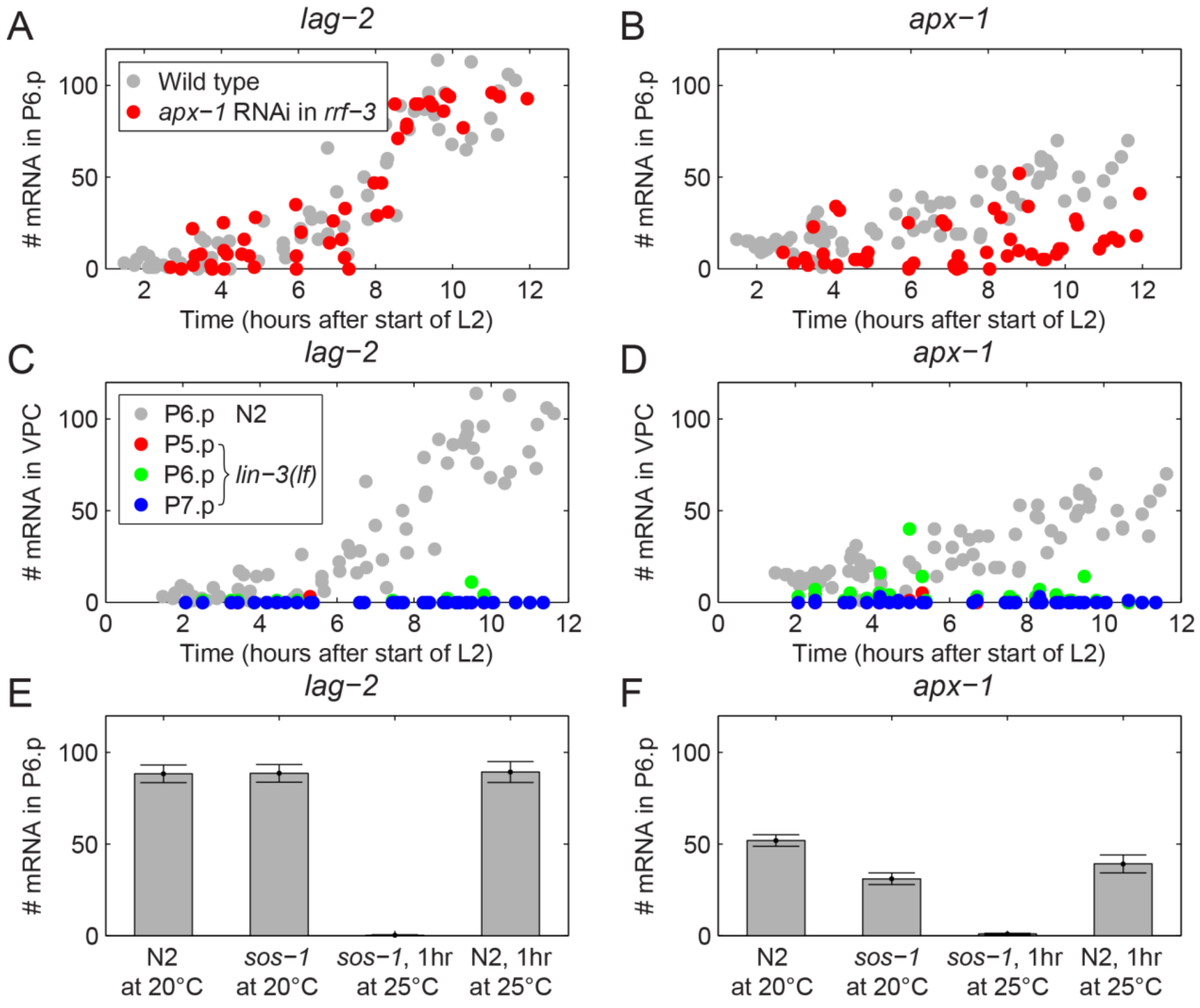

Supplementary Figure 2. **Negative controls of Notch ligand expression dynamics.**

(A) and **(B)** Expression of *lag-2* (A) and *apx-1* (B) in P6.p in wild-type animals (grey markers, *n*=73 animals) and *rrf-3* animals undergoing *apx-1* RNAi treatment (red markers, *n*=51 animals). Expression of *apx-1*, but not of *lag-2*, is reduced. **(C)** and **(D)** Expression of *lag-2* (C) and *apx-1* (D) in P(5-7).p (colored markers) in the *lin-3(e1417)* mutant, where *lin-3* expression in the AC is strongly reduced (*n*=35 animals). Expression levels in P6.p in wild-type animals are shown as a comparison (grey markers). **(E)** and **(F)** Mean expression level (grey bar) and standard error of the mean (black error bars) of *lag-2* (E) and *apx-1* (F) in P6.p obtained for wild-type and *sos-1(ts)* animals with or without a 1hr heat shock treatment. Expression levels were measured at the late induction stage, 10-12hr after the start of L2. Absence of Notch ligand expression is only observed in *sos-1(ts)* animals at the restrictive temperature, 25°C. For all experiments the data represents $n \geq 9$ animals.

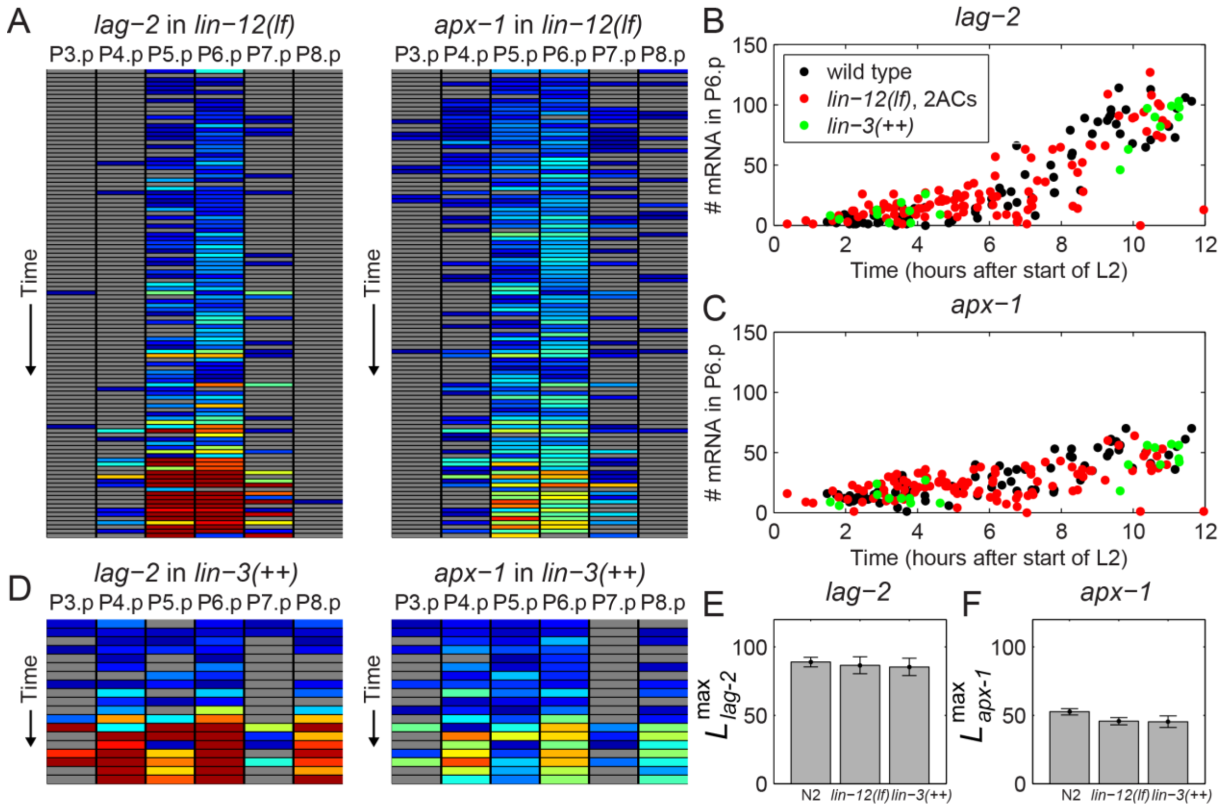

Supplementary Figure 3. **Inhibition of Notch signaling and increase of LIN-3 dosage.**

(A) Overview of *lag-2* and *apx-1* expression in the *lin-12(lf)* mutant. Columns correspond to individual VPCs. Rows represent different animals and are sorted according to increasing gonad length. Colors represent the mRNA level with the same scale as used in Fig. 2C and D in the main text. **(B)** *lag-2* and **(C)** *apx-1* mRNA levels in P6.p as a function of time in wild-type animals (n=73) and in *lin-12(lf)* (*n*=111) and *lin-3(++)* mutants (*n*=20). **(D)** Same as (A) but for the *lin-3(++)* mutant. **(E)** and **(F)** Maximally induced mRNA levels $L^{\max}_{lag-2}$ (E) and $L^{\max}_{apx-1}$ (F) for wild-type animals and for *lin-12(lf)* and *lin-3(++)* mutants. The maximally induced mRNA level is defined as the mean of the mRNA in P6.p of all animals in the late induction stage, >9 hr after the start of L2. Error bars indicate the standard error of the mean. Each experiment represents data for $n \geq 9$ animals. Even though in *lin-3(++)* animals internal Ras signaling in P6.p is expected to be hyperactivated, the measured values of the maximally induced mRNA levels in these mutants is similar to wild-type animals. This confirms our hypothesis that the measured mRNA levels in the late induction stage indeed correspond to the maximally induced mRNA levels.

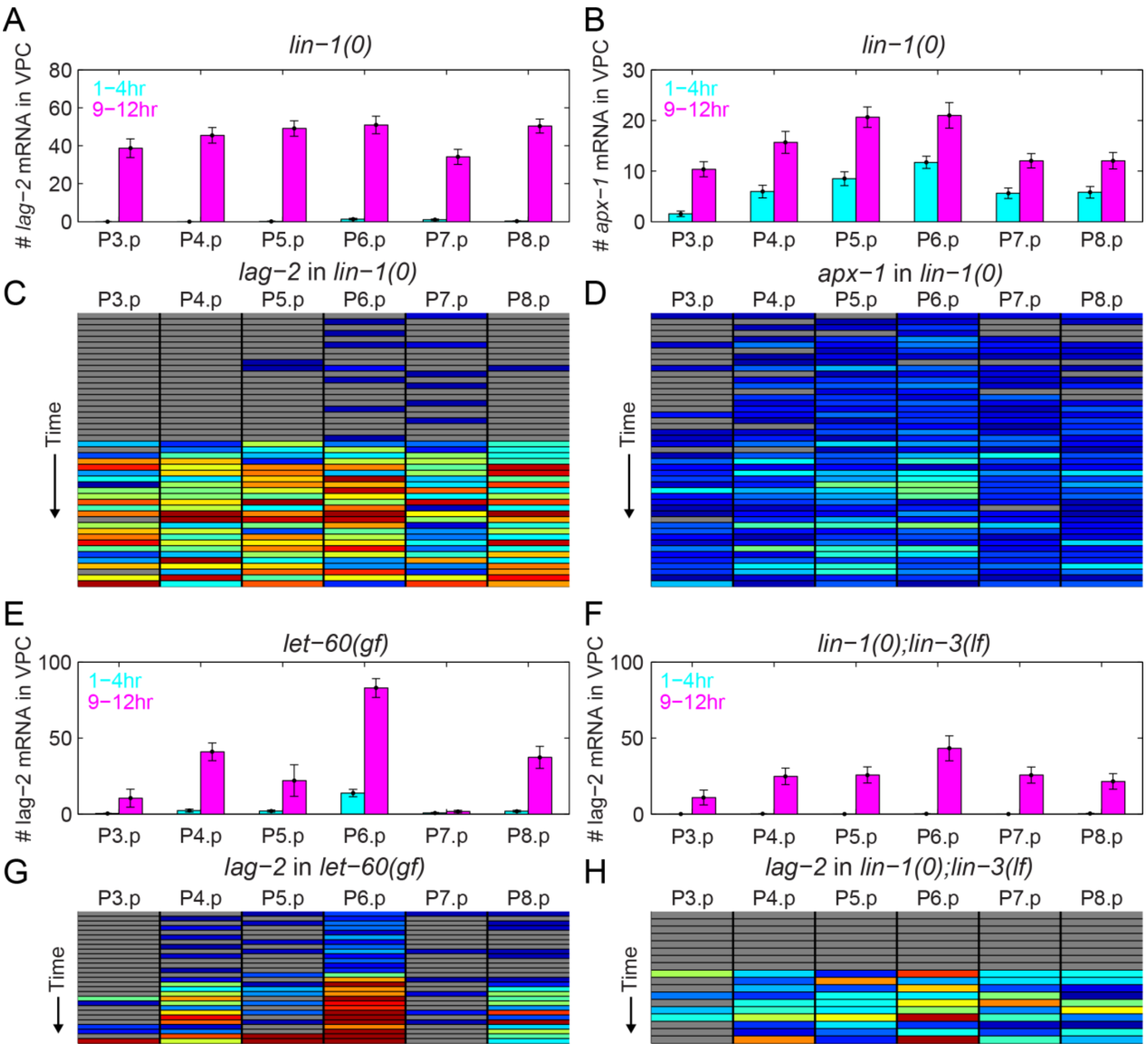

Supplementary Figure 4. **EGF/Ras signaling mutants.**

(A) Mean *lag-2* expression level (bars) and standard error of the mean (error bars) in all VPCs, P(3-8).p, in the *lin-1(0)* mutant ($n \geq 22$ animals). Shown are the expression levels at early induction (cyan bars, 1-4hr after the start of L2) and late induction (magenta bars, 9-12hr after the start of L2). **(B)** Same as (A) but for *apx-1*. **(C)** Overview of *lag-2* and *apx-1* expression in the *lin-1(0)* mutant. Columns correspond to individual VPCs. Rows represent different animals and are sorted according to increasing gonad length. Colors represent the mRNA level with the same scale as used in Fig. 2C and D in the main text. **(E)** Same as (A) but for the *let-60(gf)* mutant ($n \geq 7$ animals). **(F)** Same as (A) but for the *lin-1(0);lin-3(e1417)* mutant ($n \geq 7$ animals). **(G)** Same as (C) but for the *let-60(gf)* mutant. **(H)** Same as (C) but for the *lin-1(0);lin-3(e1417)* mutant.

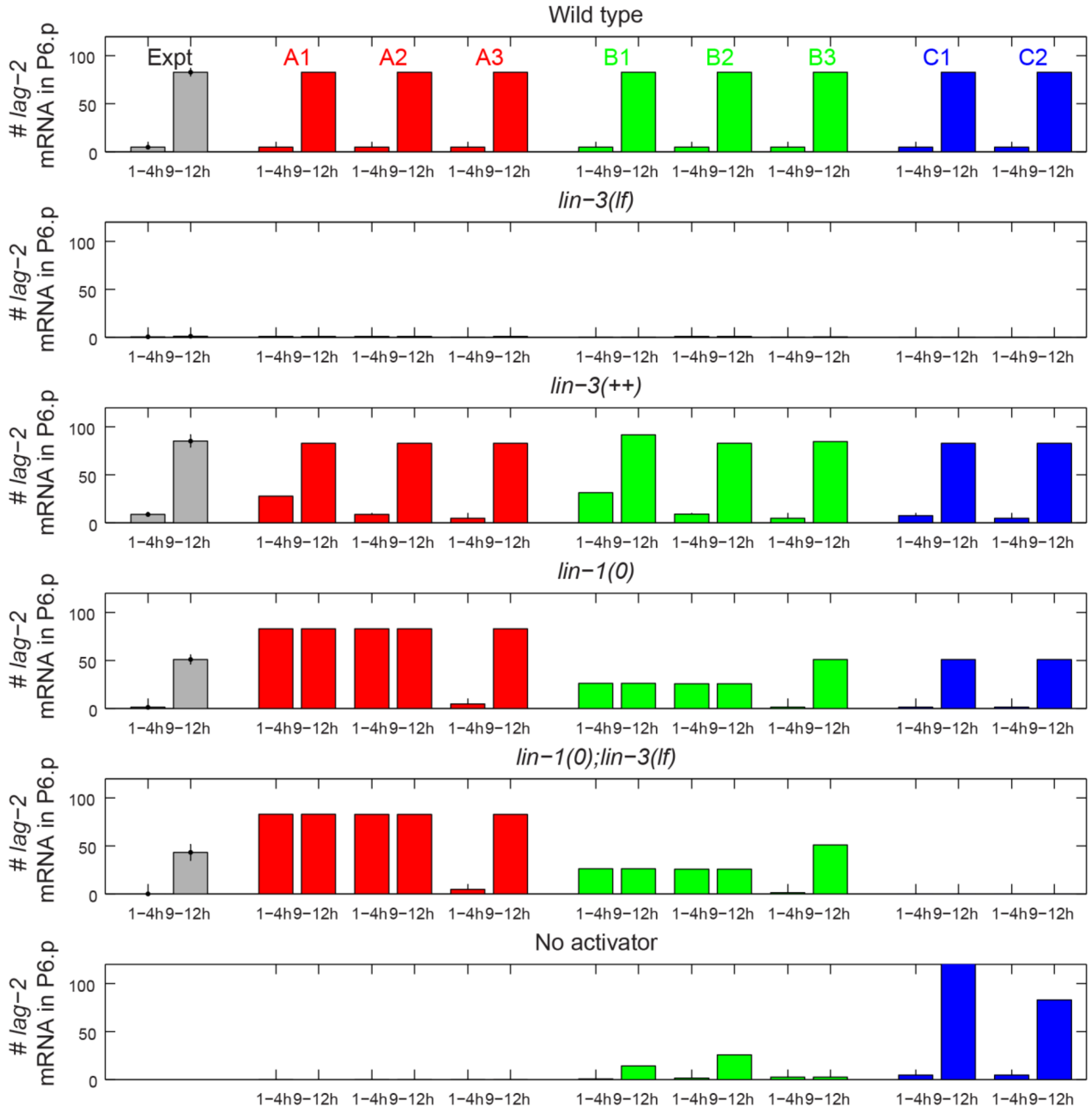

Supplementary Figure 5. **Overview of the best fits of Models A, B and C.**

Best fits of the 8 models A1-A3 (red bars), B1-B3(green bars) and C1-C2 (blue bars) to the experimental data for *lag-2* expression in P6.p in wild-type, *lin-3(e1417)*, *lin-3(++)* and *lin-1(0)* animals as well as the model predictions for the *lin-1(0);lin-3(e1417)* mutants and a hypothetical mutant lacking the activator *A*. For the experiments, mean expression level (grey bar, $n \geq 7$ animals) and standard error of the mean (black error bars) are shown. In contrast to Models A1 and B1, Model C1 is able to reproduce the experimental data for the *lin-3(++)* mutant, even though in this model the transition is due to a change in LIN-3 level. However, in contrast to Models A2-3, B2-3 and C2 this agreement only occurs for extremely precisely tuned model parameters and hence in the main text we already discard Model C1 based on lack of agreement with the *lin-3(++)* data.

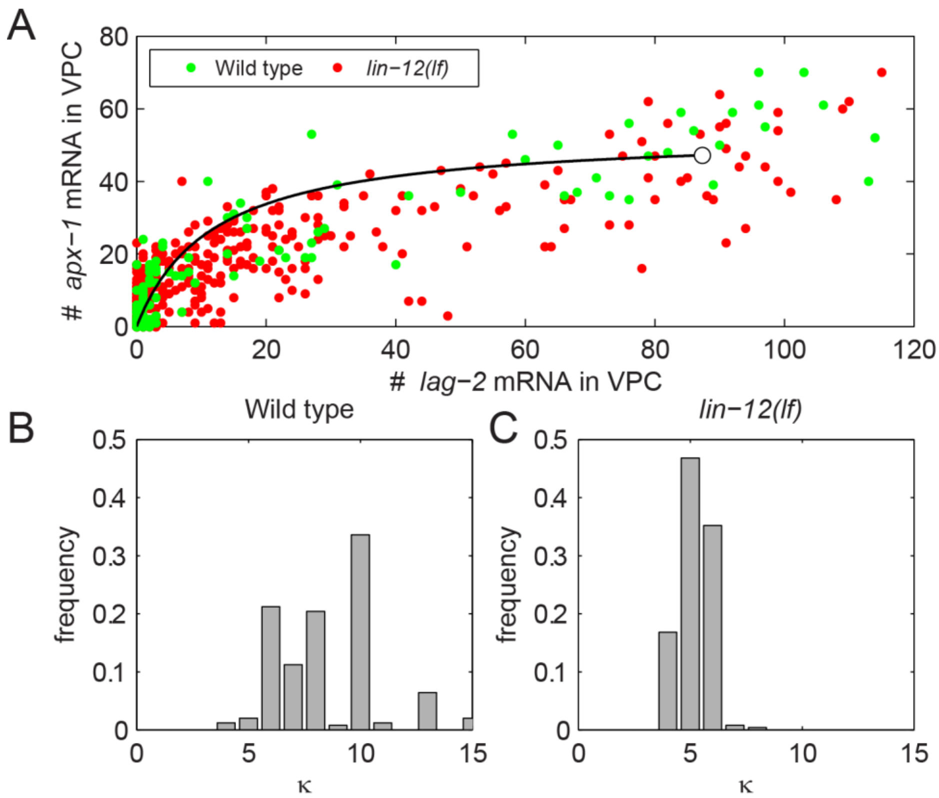

Supplementary Figure 6. **Single-cell correlation between *lag-2* and *apx-1* expression.**

(A) Single-cell correlation between *apx-1* and *lag-2* mRNA levels in P(5-7).p in wild-type animals (green, *n*=73) and P(4-8).p in *lin-12(lf)* animals (red, *n*=111). Line indicates the fit to Eq. (1) in the main text for wild-type animals with $\kappa = 8.5$. **(B)** and **(C)** Distributions obtained for κ for (B) wild-type and (C) *lin-12(lf)* animals for the bootstrap analysis described in the main text.

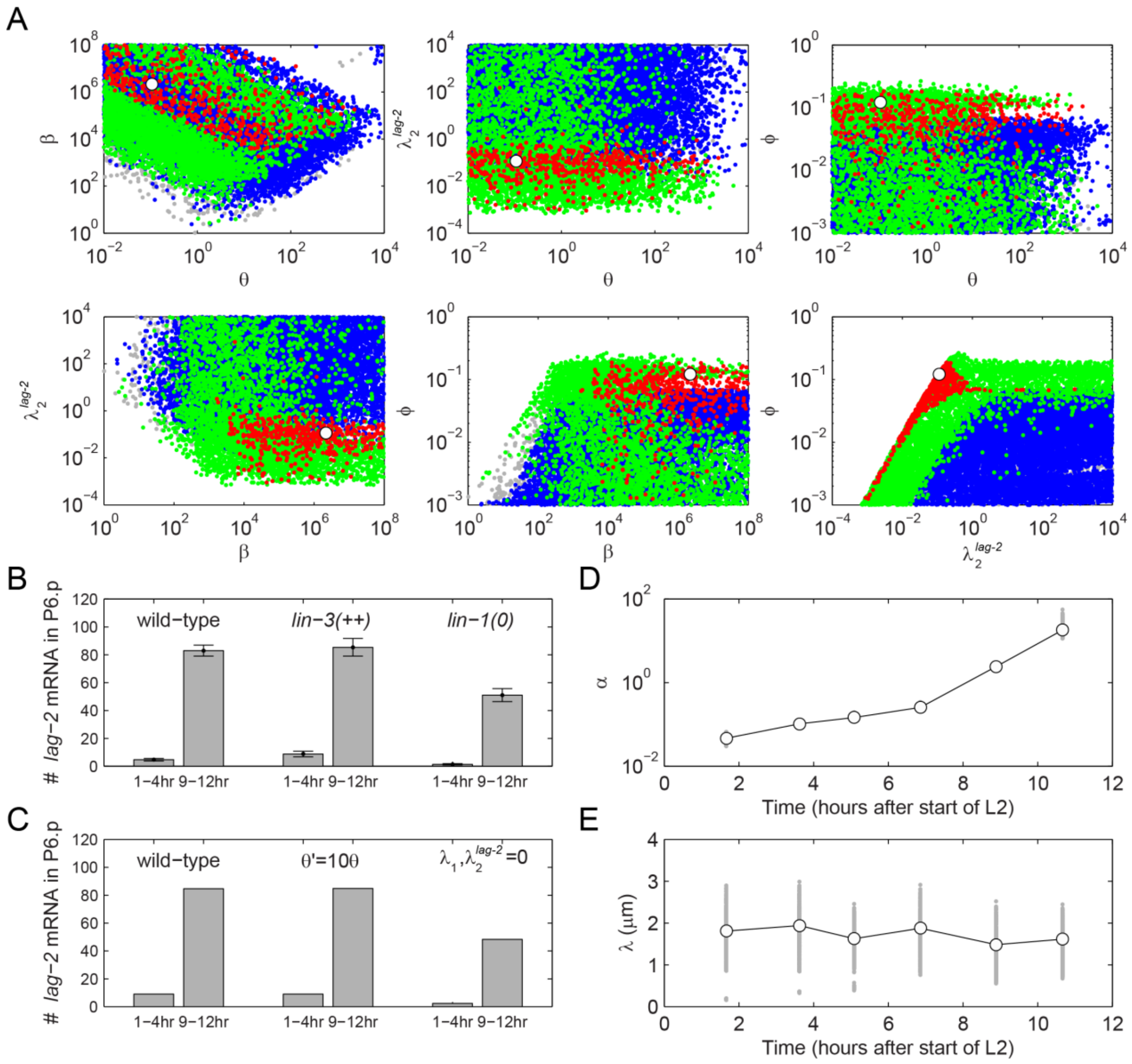

Supplementary Figure 7. **Fitting the extended Model B3 to the *lag-2* and *apx-1* expression data.**

(A) Overview of parameter combinations that yield good fits to the experimental data. Grey markers indicate parameter combinations that are good fits to the *lin-12(lf)* data, but not to the *lin-3(++)* and the *lin-1(0)* data. Blue markers indicate parameter combinations that give good fits to the *lin-12(lf)* and *lin-3(++)* data, but not to the *lin-1(0)* data. Green markers indicate parameter combinations that are good fits to the *lin-12(lf)* and the *lin-1(0)* data, but not to the *lin-3(++)* data. Finally, red markers indicate good fits to all three data sets. The overall best fit to the data, as characterized by the total RMSE, is indicated by the white marker. **(B)** and **(C)** Comparison of the transition in *lag-2* expression in P6.p between experiments (B) and the best fit (C) shown in Panel A. In panel B, grey bars indicate the mean ($n \geq 8$ animals) and the error bars indicate the standard error of the mean. **(D)** and **(E)** Time dynamics of $\alpha(t)$ (D) and $\lambda(t)$ (E) for the best fit in Panel A (white marker) and for the best 1% of fits (grey markers).

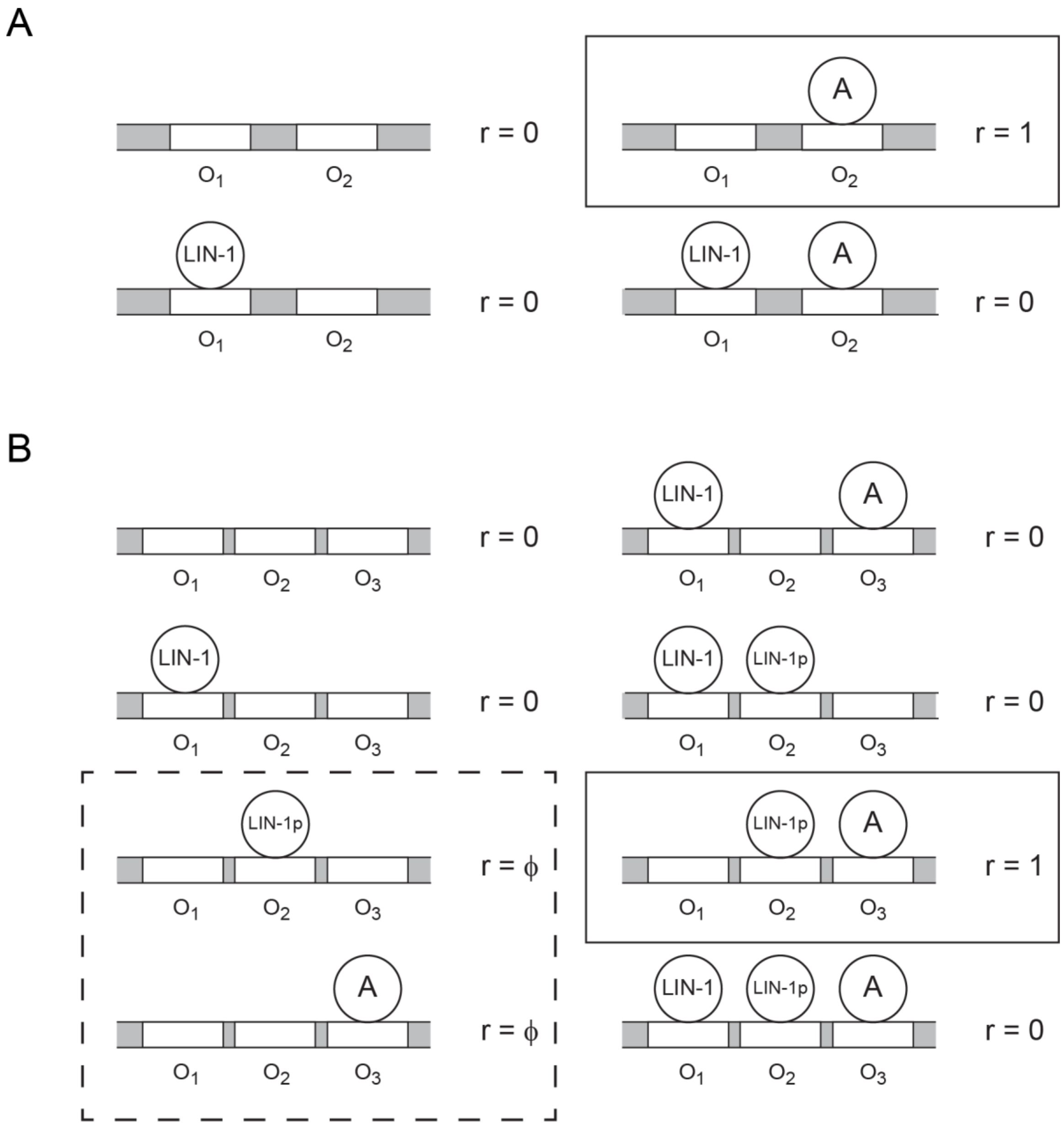

Supplementary Figure 8. **Transcription factor binding models for Models A, B and C.**

(A) Schematic description for Model A of the dependence of the *lag-2* transcription rate *r* on the binding of the repressor LIN-1 to the binding site O_1 and the activator A to the binding site O_2. Transcription only occurs, at the maximum rate *r=1*, when the activator A but not the repressor LIN-1 is bound to the promoter (solid box). **(B)** Schematic description for Models B and C of the dependence of the *lag-2* transcription rate on the binding of the repressor LIN-1 to the binding site O_1, the activator LIN-1-P to the binding site O_2 and the activator A to the binding site O_3. When both activators LIN-1-P and A are bound transcription occurs at the maximum rate *r=1* (solid box). When either LIN-1-P or A are bound, transcription occurs at a reduced rate $\phi < 1$ (dashed box).

Supplementary Note 1. LIN-3 secretion and binding to LET-23

We assume that both the LIN-3 morphogen gradient dynamics and the LIN-3 binding kinetics are in steady state. With this assumption we arrive at the following diffusion equation for the free LIN-3 concentration $l(x)$, where x is distance along the anteroposterior axis to the Anchor Cell (AC), the source of LIN-3:

$$D\partial_x^2 l(x) - \gamma l(x) - f_l l(x)\big(R_T - R(x)\big) + b_l R(x) = 0. \quad (1)$$

Here, D is the LIN-3 diffusion constant, γ is LIN-3 clearance rate, R_T is the total concentration of the EGF receptor LET-23 and f_l and b_l are the forward and backward rate of binding of LIN-3 to LET-23, respectively. In addition, secretion of LIN-3 by the AC at a rate k_p is incorporated using the boundary condition:

$$-D\partial_x l(x)|_{x=0} = k_p \quad (2)$$

The steady state equation for the concentration of the activated LIN-3-LET-23 complex, $R(x)$, given by:

$$f_l l(x)\big(R_T - R(x)\big) - b_l R(x) = 0 \quad (3)$$

Solving Eq. (3) yields:

$$R(x) = \frac{f_l l(x)}{b_l + f_l l(x)} R_T \quad (4)$$

Inserting this expression in Eq. (13) yields:

$$D\partial_x^2 l(x) - \gamma l(x) = 0. \quad (5)$$

Solving Eqs. (5) and (2) gives:

$$l(x) = \frac{k_p}{\lambda\gamma}\, e^{-|x/\lambda|} \quad (6)$$

where the decay length is given by:

$$\lambda = \sqrt{\frac{D}{\gamma}} \quad (7)$$

Using this expression, we find the following expression for $R(x)$:

$$R(x) = \frac{\theta p(x,\lambda)}{1 + \theta p(x,\lambda)} R_T \quad (8)$$

where

$$\theta = \frac{2\,k_{\mathrm{p}}}{\gamma}\frac{f_{\mathrm{l}}}{b_{\mathrm{l}}} \tag{9}$$

is a parameter that we label the LIN-3 input strength and that describes all extracellular dynamics of LIN-3 secretion and binding to the LET-23 receptor. Then, $p(x,\lambda) = \frac{1}{2\lambda}\exp(-|x/\lambda|)$ is the fraction of total free LIN-3 at position x, with $\int p(x,\lambda)dx = 1$.

Supplementary Note 2. Induction of Ras signaling by activated LET-23

The Vulva Precursor Cell (VPC) integrates the concentration of activated LET-23 over the entire basolateral surface of the cell. Hence, in our model the Ras signaling strength $\sigma(x_i)$ for a VPC at distance x_i to the AC is given by the total concentration of activated LIN-3-LET-23 complexes integrated over the body of the VPC:

$$\sigma(x) = \int_{x-{L_{\mathrm{c}}}/_2}^{x+{L_{\mathrm{c}}}/_2} R(x')dx' \tag{10}$$

where L_{C} is the length of the VPC body along the A-P axis. Using Eq. (8) we arrive at the following expression of the Ras signaling strength:

$$\sigma(x) = R_{\mathrm{T}}E(x,\lambda,\theta) = R_{\mathrm{T}}\int_{x-{L_{\mathrm{c}}}/_2}^{x+{L_{\mathrm{c}}}/_2} \frac{\phi p(x',\lambda)}{1+\phi p(x',\lambda)}dx' \tag{11}$$

We calculate $E(x,\lambda,\phi)$ analytically using the expression:

$$E(x,\lambda,\phi) = \begin{cases} F(b)-F(a), & a,b \geq 0 \\ F(b)-2F(0)+F(-a), & b \geq 0, a < 0 \\ F(-a)-F(-b), & a,b < 0 \end{cases} \tag{12}$$

where $a = x - L_{\mathrm{C}}/2$, $b = x + L_{\mathrm{C}}/2$ and $F(x) = x - \lambda\ln\left(e^{x/\lambda} + \frac{\phi}{2\lambda}\right)$.

Supplementary Note 3. Mathematical modeling of Notch ligand expression dynamics

Model A

We assume that LIN-1 is phosphorylated in response to Ras signaling with rate $f_{\mathrm{L}} \cdot \sigma$, with σ given by Eq. (11), and spontaneously dephosphorylates at rate b_{L}. The activator A is converted from its inactive form A* to the active form A with forward rate f_{A} and converted back at rate b_{A}. On the timescale of induction these reactions are in steady state, leading to the following equations for the activation of the transcription factors LIN-1 and A:

$$[\mathrm{L_p}] = K_{\mathrm{L}}\sigma[\mathrm{L}] \tag{13}$$

$$[\mathrm{A}] = K_{\mathrm{A}}[\mathrm{A}^*] \tag{14}$$

where $[\mathrm{L_p}]$ indicates the concentration of LIN-1-P, [L] the concentration of LIN-1, $K_{\mathrm{L}} = f_{\mathrm{L}}/b_{\mathrm{L}}$ and $K_{\mathrm{A}} = f_{\mathrm{A}}/b_{\mathrm{A}}$.

We assumed that the transcription rate of Notch ligand mRNA depends only on the probability of the transcription factors LIN-1 and A being bound to their respective binding sites $\mathrm{O_1}$ and $\mathrm{O_2}$ according to Fig. 8, constituting a so-called "thermodynamic model" of gene expression. In this picture, the steady state binding probabilities of the transcription factors LIN-1 and A are given by:

$$[\mathrm{O_1L}] = \widetilde{K}_{\mathrm{L}}[\mathrm{O_1}][\mathrm{L}] \tag{15}$$

$$[\mathrm{O_2A}] = \widetilde{K}_{A}[\mathrm{O_2}][\mathrm{A}] \tag{16}$$

where $[\mathrm{O_1L}]$ and $[\mathrm{O_2A}]$ are the concentration of LIN-1 and A at their binding sites and K_L and K_A are their respective binding constants. Transcription occurs at a rate $r = 1$ when A but not LIN-1 is bound, reflecting their respective roles as activator and repressor. This gives rise to the following expression of the transcription rate:

$$r = [O_2A](1 - [O_1L]) \tag{17}$$

To calculate an explicit expression for the transcription rate, we use the following conservation equations:

$$[L] + [L_p] = L_T, \qquad [A^*] + [A] = A_T \tag{18}$$

$$[O_1] + [O_1L] = 1, \qquad [O_2] + [O_2A] = 1 \tag{19}$$

where L_T and A_T are the total concentrations of LIN-1 and A in the VPC. Using the above equations, we calculated the following expressions for the concentration of the different transcription factor species LIN-1, LIN-1-P and A:

$$[L] = L_T\frac{1}{1 + K_L\sigma}, \qquad [L_p] = L_T\frac{K_L\sigma}{1 + K_L\sigma} \tag{20}$$

$$[A] = A_T\frac{K_A}{1 + K_A} \tag{21}$$

and the following expressions for the concentration of unbound promoter binding sites:

$$\left(1 + \widetilde{K}_L[L]\right)[O_1] = 1 \tag{22}$$

$$\left(1 + \widetilde{K}_A[A]\right)[O_2] = 1 \tag{23}$$

yielding the following expressions for the concentration of bound promoter binding sites O_1 and O_2:

$$[O_1L] = \frac{\widetilde{K}_L L_T \frac{1}{1 + K_L\sigma}}{1 + \widetilde{K}_L L_T \frac{1}{1 + K_L\sigma}} \tag{24}$$

$$[O_2A] = \frac{\widetilde{K}_A A_T \frac{K_A}{1 + K_A}}{1 + \widetilde{K}_A A_T \frac{K_A}{1 + K_A}} \tag{25}$$

This results in the following expression for the transcription rate for model A:

$$r = \frac{\alpha}{\left(1 + \lambda_1 \frac{1}{1 + s}\right)(1 + \alpha)} \tag{26}$$

where:

$$\lambda_1 = \widetilde{K}_L L_T, \qquad \alpha = \widetilde{K}_A A_T \frac{K_A}{1 + K_A}, \qquad s = K_L\sigma \tag{27}$$

Model B

In Model B, LIN-1 and A still act as a repressor and an activator, as in Model A, but now in addition LIN-1-P also activates Notch ligand expression (See Fig. 8). The concentrations of LIN-1, LIN-1-P and A are given by Eqs. (13)-(14). Similar to Model A, the steady-state concentration of transcription factors bound to their promoter binding sites are given by:

$$[O_1L] = \widetilde{K}_L[O_1][L] \tag{28}$$

$$[O_2L_p] = \widetilde{K}_{L_p}[O_2][L_p] \tag{29}$$

$$[O_3A] = \widetilde{K}_A[O_3][A] \tag{30}$$

We assumed that no transcription occurred when the repressor LIN-1 was bound (Fig. S8, $r = 0$), that transcription occurred at a low level $\phi < 1$ when either LIN-1-P or A were bound and at the maximum level $r = 1$ when both LIN-1-P and A were bound. This gives rise to the following expression for the transcription rate:

$$r = 1 \cdot p_{high} + \phi \cdot p_{low} \tag{31}$$

where

$$p_{low} = ([O_2L_P](1 - [O_3A]) + (1 - [O_2L_P])[O_3A])(1 - [O_1L]) \tag{32}$$

and

$$p_{high} = [O_2 L_P][O_3 A](1 - [O_1 L]) \tag{33}$$

For the concentration of unbound promoters we have the following expressions:

$$\left(1 + \widetilde{K}_L [L]\right)[O_1] = 1 \tag{34}$$

$$\left(1 + \widetilde{K}_L [L_P]\right)[O_2] = 1 \tag{35}$$

$$\left(1 + \widetilde{K}_A [A]\right)[O_3] = 1 \tag{36}$$

leading to the following expression for the transcription rate for Model B:

$$r = \frac{\alpha\lambda_2 \frac{s}{1+s} + \phi(\alpha + \lambda_2 \frac{s}{1+s})}{\left(1 + \lambda_1 \frac{1}{1+s}\right)\left(1 + \lambda_2 \frac{s}{1+s}\right)(1+\alpha)} \tag{37}$$

where:

$$\lambda_1 = \widetilde{K}_L L_T, \qquad \lambda_2 = \widetilde{K}_{L_p} L_T, \qquad \alpha = \widetilde{K}_A A_T \frac{K_A}{1 + K_A}, \qquad s = K_L \sigma \tag{38}$$

Model C

Model C is very similar to Model B, with the sole change that now the conversion of the activator A from its inactive to its active form is dependen on Ras signaling, i.e. proceeds with a forward rate $f_A \cdot \sigma$. This gives rise to the following modified expression for the activation of the transcription factors LIN-1 and A:

$$[L_p] = K_L \sigma [L] \tag{39}$$

$$[A] = K_A \sigma [A^*] \tag{40}$$

Following the approach for Model B, we find the following expression for the transcription rate for Model C:

$$r = \frac{\alpha\lambda_2 \left(\frac{s}{1+s}\right)^2 + \phi(\alpha + \lambda_2)\left(\frac{s}{1+s}\right)}{\left(1 + \lambda_1 \frac{1}{1+s}\right)\left(1 + \lambda_2 \frac{s}{1+s}\right)\left(1 + \alpha \frac{s}{1+s}\right)} \tag{41}$$

where:

$$\lambda_1 = \widetilde{K}_L L_T, \qquad \lambda_2 = \widetilde{K}_{L_p} L_T, \qquad \alpha = \widetilde{K}_A A_T, \qquad s = K_L \sigma \tag{42}$$

Supplementary Note 4. Summary of Models A, B and C for wild-type and mutants

In the table below, we summarize the expressions for the Notch ligand transcription rate in Models A, B and C. We also show the expressions for the transcription rate in the *lin-3(0)* mutant, corresponding to $s = 0$, as well as in the *lin-1(0)* mutant, corresponding to $\lambda_1, \lambda_2 = 0$ and the mutant lacking the activator A, corresponding to $\alpha = 0$:

	Model A	Model B	Model C
WT	$r = \frac{\alpha}{\left(1+\lambda_1 \frac{1}{1+s}\right)(1+\alpha)}$	$r = \frac{\alpha\lambda_2 \frac{s}{1+s} + \phi(\alpha + \lambda_2 \frac{s}{1+s})}{\left(1+\lambda_1 \frac{1}{1+s}\right)\left(1+\lambda_2 \frac{s}{1+s}\right)(1+\alpha)}$	$r = \frac{\alpha\lambda_2 \left(\frac{s}{1+s}\right)^2 + \phi(\alpha+\lambda_2)\left(\frac{s}{1+s}\right)}{\left(1+\lambda_1 \frac{1}{1+s}\right)\left(1+\lambda_2 \frac{s}{1+s}\right)\left(1+\alpha \frac{s}{1+s}\right)}$
lin-3(0)	$r = \frac{\alpha}{(1+\lambda_1)(1+\alpha)}$	$r = \frac{\phi\alpha}{(1+\lambda_1)(1+\alpha)}$	$r = 0$
lin-1(0)	$r = \frac{\alpha}{1+\alpha}$	$r = \frac{\phi\alpha}{1+\alpha}$	$r = \frac{\phi\alpha\left(\frac{s}{1+s}\right)}{1+\alpha \frac{s}{1+s}}$
A(0)	$r = 0$	$r = \frac{\phi\lambda_2 \frac{s}{1+s}}{\left(1+\lambda_1 \frac{1}{1+s}\right)\left(1+\lambda_2 \frac{s}{1+s}\right)}$	$r = \frac{\phi\lambda_2 \frac{s}{1+s}}{\left(1+\lambda_1 \frac{1}{1+s}\right)\left(1+\lambda_2 \frac{s}{1+s}\right)}$

Supplementary Note 5. Overview of parameters in Models A, B and C

Parameter	Definition
$s = \beta\, E(x, \lambda, \theta)$	Ras signaling strength
x	Distance of VPC to AC
λ	LIN-3 gradient decay length
θ	LIN-3 input strength
β	Sensitivity of Ras signaling pathway to external LIN-3
λ_1	Binding affinity of repressive LIN-1 to *lag-2* promoter
λ_2	Binding affinity of activating LIN-1-P to *lag-2* promoter
ϕ	Transcription rate of either LIN-1-P or A bound relative to transcription rate when both LIN-1-P and A bound
α	Binding affinity/amount of activator A

Supplementary Note 6. Fitting models A, B and C to *lin-3(++)* and *lin-1(0)* mutant data

To examine how the observed transition in Notch ligand expression is generated on the network level, we tested, for each of the Models A, B and C, three potential underlying mechanisms: first, the case where the transition was driven by a change in external LIN-3 level (Models A1, B1 and C1 in Fig. 5A in the main text). Second, the case where the transition was driven by a change in sensitivity of the Ras pathway to the external LIN-3 signal (Models A2, B2 and C2). Third, the case where the transition was driven by a change in the amount of activator A (Models A3 and B3). We implemented this in the context of the models A, B and C by assuming that all parameters except for a single parameter are constant in time. For Models A1, B1 and C1, then the only parameter changing in time is $\theta(t)$. For Models A2, B2 and C2 the only parameter changing in time is $\beta(t)$, rewriting Eqs. (11) and the expression for s in Eqs. (27) or (38) so that $s = \beta\, E(x, \lambda, \theta)$, with $\beta = K_L\, R_T$. Finally, for Models A3 and B3 the only parameter changing in time is $\alpha(t)$. Below, as an illustration we will outline the procedure for fitting Model B3, where α varies in time, to the *lin-3(++)* and *lin-1(0)* data for *lag-2* (Fig. 5B-D in the main text). The procedure for fitting the other models is identical, apart from the fact that a different time-dependent parameter is used.

We perform our fitting procedure under the constraint that the model should reproduce the wild-type data for *lag-2* and show no induction of *lag-2* expression in the absence of LIN-3, i.e. $s = 0$. The first constraint is given by:

$$\frac{r(\alpha = \alpha_{early})}{r(\alpha = \alpha_{late})} = \frac{L^{early}_{lag-2}}{L^{late}_{lag-2}} \tag{43}$$

where α_{early} and α_{late} are the values for $\alpha(t)$ at the early (1-4hr) and late (9-12hr) induction and L^{early}_{lag-2} and L^{late}_{lag-2} are the mean *lag-2* mRNA levels for early and late induction (Fig. 5B in the main text). In Model B3, we still have basal induction for $s = 0$, with the magnitude depending on the value of α and λ_1. Here, we constrain λ_1 so that in the absence of LIN-3 the *lag-2* expression level is less than 1% of the fully induced level:

$$r(\alpha = \alpha_{late}, s = 0) \leq f \cdot r(\alpha = \alpha_{late}) \tag{44}$$

with $f = 0.01$.

As an initial guess for the fitting procedure, we picked values for the parameters $\lambda, \theta, \beta, \lambda_1, \lambda_2, \phi, \alpha_{early}$ and α_{late}, so that the constraints in Eqs. (43)and (44) were satisfied. We then fit the model to the *lin-3(++)* and *lin-1(0)* data using the Sequential Least Squares Programming (SLSQP) constrained optimization algorithm to minimize the following error function:

$$\epsilon = \sum_{m=\{\text{lin-3(++)},\text{lin-1(0)}\}} \sum_{t=\{\text{early},\text{late}\}} \left[r_m\big(\alpha(t)\big) - \frac{L^{m}_{lag-2}(t)}{L^{WT,late}_{lag-2}} \right]^2 \tag{45}$$

where the index m indicates the different mutants, t indicates the different time points, $L_{lag-2}^{m}(t)$ is the mean *lag-2* mRNA level of mutant m at time point t (Fig. 5C and D in the main text) and $L_{lag-2}^{WT,late}$ is the *lag-2* mRNA level at the late induction timepoint. The *lag-2* transcription rate for the *lin-3(++)* mutant is given by $r_{\text{lin-3(++)}} = r(\theta' = 10\theta)$, corresponding to a tenfold increase in LIN-3 dosage. The *lag-2* transcription rate for the *lin-1(0)* mutant is given by $r_{\text{lin-1(0)}} = r(\lambda_1 = 0, \lambda_2 = 0)$, corresponding to the complete absence of LIN-1 and LIN-1-P. The constrained optimization algorithm ensured that the constraints in Eqs. (43) and (44) are maintained throughout the fitting procedure. In the case of Model B3 we found that we could simultaneously fit the wild-type, *lin-3(++)* and *lin-1(0)* mutant data (Fig. 5), with many parameter combinations giving equally good fits. We interpret this as Model B3 being consistent with the experimental data. However, for the other models we found that we were unable to fit the data for wild-type animals and one or both mutants simultaneously. We interpreted this as those models being incomplete or incorrect.

Supplementary Note 7. Regulation of *lag-2* and *apx-1* expression in model B3

The expression for the transcription rate for Model B3 can rewritten as:

$$r = \frac{r_0 + r_\infty \alpha(t)}{1 + \alpha(t)} \tag{46}$$

where:

$$r_0 = \frac{\phi \lambda_2 \frac{s}{1+s}}{\left(1 + \lambda_1 \frac{1}{1+s}\right)\left(1 + \lambda_2 \frac{s}{1+s}\right)}, r_\infty = \frac{\phi + \lambda_2 \frac{s}{1+s}}{\left(1 + \lambda_1 \frac{1}{1+s}\right)\left(1 + \lambda_2 \frac{s}{1+s}\right)} \tag{47}$$

where $\alpha(t)$ is the only time-dependent variable. The mRNA level L is given by $L = r/d$, where d is the mRNA degradation rate. For full induction, $\alpha \to \infty$, the expression for the maximally induced mRNA level is given by $L^m = r_\infty/d$, whereas in the absence of activator A, $\alpha = 0$, the basal mRNA level is given by $L^b = r_0/d$. We assumed that the only parameters that can control the difference in expression patterns observed between *lag-2* and *apx-1* are those governing the interaction between LIN-1, LIN-1-P and A and their binding sites in the *lag-2* and *apx-1* promoters, i.e. $\lambda_1, \lambda_2, \phi$ and α. However, λ_1, λ_2 and ϕ only influence the basal, r_0, and maximum expression level, r_∞, not how the expression level increases as a function of the amount of activator. Hence, to capture the difference in time dynamics between *lag-2* and *apx-1* expression we have to assume that the binding of A to the *lag-2* and *apx-1* promoter occurs with different affinity. For this we use the simplest assumption that $\alpha_i(t) = C_i \cdot \alpha(t)$ for each Notch ligand *i*. Finally, to capture the difference in maximum expression level between *lag-2* and *apx-1*, we use the simplest assumption that only one parameter, which we choose to be the LIN-1-P

binding rate λ_2, differs between *lag-2* and *apx-1*. Hence, we need to consider two values λ_2^i for each Notch ligand *i,* with the values of the remaining parameters λ_1 and ϕ the same for both the *lag-2* and *apx-1* promoter.

Supplementary Note 8. Single-cell correlation between *lag-2* and *apx-1* expression

Using Eq. (46), we can write the expression level L_i of Notch ligand i as:

$$L_i(t) = \frac{L_i^b + L_i^m C_i a(t)}{1 + C_i a(t)} \tag{48}$$

Solving for $\alpha(t)$ gives:

$$a(t) = \frac{L_i(t) - L_i^b}{\theta_i(L_i^m - L_i(t))} \tag{49}$$

If the two ligands i and j are coregulated by same activator A, as we assume is the case for *lag-2* and *apx-1*, we can insert the expression for $\alpha(t)$ into the expression for $L_j(t)$, yielding:

$$L_j(t) = \frac{L_j^b L_i^m - \kappa L_i^b L_j^m - \left(L_j^b - \kappa L_j^m\right) L_i(t)}{L_i^m - \kappa L_i^b + (\kappa - 1) L_i(t)} \tag{50}$$

Experimentally, we observe no expression of *lag-2* and *apx-1* in VPCs at the start of vulva induction, i.e. $L_i(t) = 0$ for *i={lag-2,apx-1}*. Hence, the parameters , λ_1, λ_2^i and ϕ are tuned so that the basal expression levels vanish, $L_i^b, L_j^b = 0$, and we have:

$$L_j(t) = L_j^m \frac{\kappa L_i(t)}{L_i^m + (\kappa - 1) L_i(t)} \tag{51}$$

where we have $\kappa = C_j / C_i$, corresponding to Eq. 1 in the main text. In the context of Model B3, we interpret κ as follows: the conversion of active to inactive A, as given by the equilibrium constant K_A, is the only process that depends on time. At the same time, the binding affinity of active A is constant in time, but different for each promoter. With these assumptions and using the expression for α in Eq. (38), we get the following expression for $\alpha(t)$:

$$\alpha(t) = \widetilde{K}_A^i A_T \frac{K_A(t)}{1 + K_A(t)} \tag{52}$$

Thus, in this interpretation $\kappa = \tilde{K}_A^j / \tilde{K}_A^i$, which is the ratio between the thresholds for induction of Notch ligand *i* and *j* by the activator A. Here, $\kappa > 1$ would mean that Notch ligand *i* has a lower threshold to induction by Ras signaling than Notch ligand *j*.

Finally, we note that the maximally induced mRNA level L_i^m in a specific VPC depends on the Ras signaling strength s and hence on the distance of the VPC to the AC. This means that Eq. (51) is only expected to hold when we use the value of L_i^m measured in that particular VPC. In Fig. 6 in the main text, we use for L_i^m the maximally induced *lag-2* and *apx-1* mRNA levels observed in P6.p. Hence, a priori it is not expected that the single-cell correlation curve between *lag-2* and *apx-1* expression for cells other than P6.p should collapse on the same curve. Still, we find excellent overlap between curves for P6.p in wild-type animals and P4.p, P5.p and P6.p in *lin-12(lf)* animals. However, this could be responsible for the relative deviation from the theoretical curve observed for P7.p cells in the *lin-12(lf)* mutant.

Supplementary Note 9. Overview of parameters of the extended Model B3

Parameter	Definition
$s = \beta\, E(x, \lambda(t), \theta)$	Ras signaling strength
x	Distance of VPC to AC
$\lambda(t)$	LIN-3 gradient decay length, time-dependent
θ	LIN-3 input strength
β	Sensitivity of Ras signaling pathway to external LIN-3
λ_1	Binding affinity of repressive LIN-1 to *lag-2* and *apx-1* promoter
λ_2^i	Binding affinity of activating LIN-1-P to promoter of Notch ligand *i*
ϕ	Transcription rate of either LIN-1-P or A bound relative to transcription rate when both LIN-1-P and A bound
$\alpha_i(t)$	Binding affinity of activator A to the promoter of Notch ligand *i*, time-dependent
κ	Ratio of binding affinitys of activator A for the *lag-2* and *apx-1* promoter, $\alpha_{apx-1} = \kappa\, \alpha_{lag-2}$
d	*lag-2* and *apx-1* mRNA degradation rate

Supplementary Note 10. Fitting Model B3 to experimental *lag-2* and *apx-1* expression data

We found that the extended Model B3 has four independent global parameters: θ, β, ϕ and λ_2^{lag-2}. In addition, for each time point in Fig. 4A and B in the main text the model has two additional independent parameters, $\lambda(t)$ and $\alpha(t)$. The remaining parameters

$\lambda_1, \lambda_2^{apx-1}$ and d are fixed by the experimental data in the following way: no induction of Notch ligand expression should occur in the absence of LIN-3 or far away from the AC, i.e. when $s = 0$, even when A is fully activated, i.e. $\alpha \to \infty$. This means that:

$$\lim_{\alpha\to\infty} \frac{L_{lag-2}(s=0)}{L_{lag-2}^m} = \lim_{\alpha\to\infty} \frac{r_\infty^{lag-2}(s=0)}{r_\infty^{lag-2}} = f \tag{53}$$

where we use $f = 1 \cdot 10^{-3}$. Equation (53) uniquely fixes the value of λ_1. Because *lag-2* is more highly expressed than *apx-1* this value of λ_1 also ensures no *apx-1* expression occurs in the absence of LIN-3. However, not all combinations of θ, β, ϕ and λ_2^{lag-2} allow for a value of λ_1 that satisfies Eq. (53). This only occurs when the following condition is met:

$$\frac{1}{1+s} \frac{\phi\left(1+\lambda_2^{\mathrm{lag-2}} \frac{s}{1+s}\right)}{\phi+\lambda_2^{\mathrm{lag-2}} \frac{s}{1+s}} > f \tag{54}$$

The value of λ_2^{apx-1} is fixed by the constraint that the model is able to reproduce the fully induced *apx-1* expression level L_{apx-1}^m:

$$\frac{r_\infty^{apx-1}}{r_\infty^{lag-2}} = \frac{L_{apx-1}^m}{L_{lag-2}^m} \tag{55}$$

However, also in this case not every combination of θ, β, ϕ and λ_2^{lag-2} allows for a value of λ_2^{apx-1} that satisfies Eq. (55). This only occurs when

$$\frac{\phi\left(1+\lambda_2^{\mathrm{lag-2}} \frac{s}{1+s}\right)}{\phi+\lambda_2^{\mathrm{lag-2}} \frac{s}{1+s}} < \frac{L_{apx-1}^m}{L_{lag-2}^m} \tag{56}$$

and

$$\frac{1+\lambda_2^{\mathrm{lag-2}} \frac{s}{1+s}}{\phi+\lambda_2^{\mathrm{lag-2}} \frac{s}{1+s}} \geq \frac{L_{apx-1}^m}{L_{lag-2}^m} \tag{57}$$

are both satisfied.

We generated $5 \cdot 10^4$ combinations of the parameters θ, β, ϕ and λ_2^{lag-2}, as outlined in the Methods section in the main text, that satisfy the above criteria. Then for each time point in Fig. 4A and B, we found values of $\lambda(t)$ and $\alpha(t)$ that minimize the SSE between the spatial expression profile predicted by Model B3 for *lag-2* and *apx-1* expression and the experimental data in Fig. 4A and B. In addition, we calculated for each parameter combination the SSE with respect to the *lag-2* expression dynamics in the *lin-3(++)* and *lin-1(0)* mutants, as outlined in the section 'Fitting models A, B and C to lin-3(++) and lin-1(0) mutant data'.
